# Psycho-cardiovascular interaction network in cardiovascular health and disease: signaling pathways and molecular mechanisms

**DOI:** 10.1093/pcmedi/pbag025

**Published:** 2026-09-01

**Authors:** Zexi Li, Fanghui Li, Chunyi Yan, Lianrui Xiang, Aobo Gong, Ying Cao, Yifan Zhou, Wei Zhang, Rui Zeng

**Affiliations:** Department of Cardiology, West China Hospital, Sichuan University, Chengdu 610041, China; Department of Cardiology, West China Hospital, Sichuan University, Chengdu 610041, China; Department of Pediatric Cardiology, West China Second University Hospital, Sichuan University, Chengdu 610066, China; Public Service and Development Office, West China Second University Hospital, Sichuan University, Chengdu 610066, China; Department of Cardiology, West China Hospital, Sichuan University, Chengdu 610041, China; Department of Cardiology, West China Hospital, Sichuan University, Chengdu 610041, China; Department of Cardiology, West China Hospital, Sichuan University, Chengdu 610041, China; Mental Health Center and Institute of Psychiatry, West China Hospital, Sichuan University, Chengdu 610041, China; Department of Cardiology, West China Hospital, Sichuan University, Chengdu 610041, China

**Keywords:** psycho-cardiovascular interactions, brain–heart axis, cardiovascular disease, mental disorders, signaling pathways, precision medicine

## Abstract

Extensive evidence indicates a strong association between psychological and psychiatric factors and cardiovascular disease (CVD). Psychological and psychiatric factors, including depression, anxiety, and chronic stress, are associated with an increased risk of CVD and adverse cardiovascular outcomes. Furthermore, this association is bidirectional, as patients with CVD have a notably higher risk of developing comorbid mental disorders. We propose the psycho-cardiovascular interaction network (PCIN) to elucidate the multilevel integrated regulatory mechanisms linking psychological status and cardiovascular function. In this review, we synthesize epidemiologic evidence, summarize the major mechanisms through which these factors may influence cardiovascular function, and focus on key signaling pathways within the PCIN, including BDNF, PROK/PKR, MAPK, 5-HT/5-HT_2_A-R, and NLRP3 inflammasome pathways, which may mediate bidirectional brain–heart communication and mental disorder–CVD comorbidity. On the basis of the PCIN framework, we discuss integrated intervention strategies, including nonpharmacological approaches and molecularly targeted therapies for brain–heart axis-informed management. Future research should focus on the translational potential of psycho-cardiovascular regulatory molecules as therapeutic targets and candidate biomarkers, and high-quality studies are urgently needed to elucidate the long-term effects of psychological interventions on cardiovascular health outcomes and their molecular mechanisms. In addition, integrating psychosocial phenotypes with molecular biomarkers may facilitate individualized risk assessment and inform brain–heart axis-based prevention and therapeutic strategies.

## Graphical Abstract

**Graphical Abstract:**
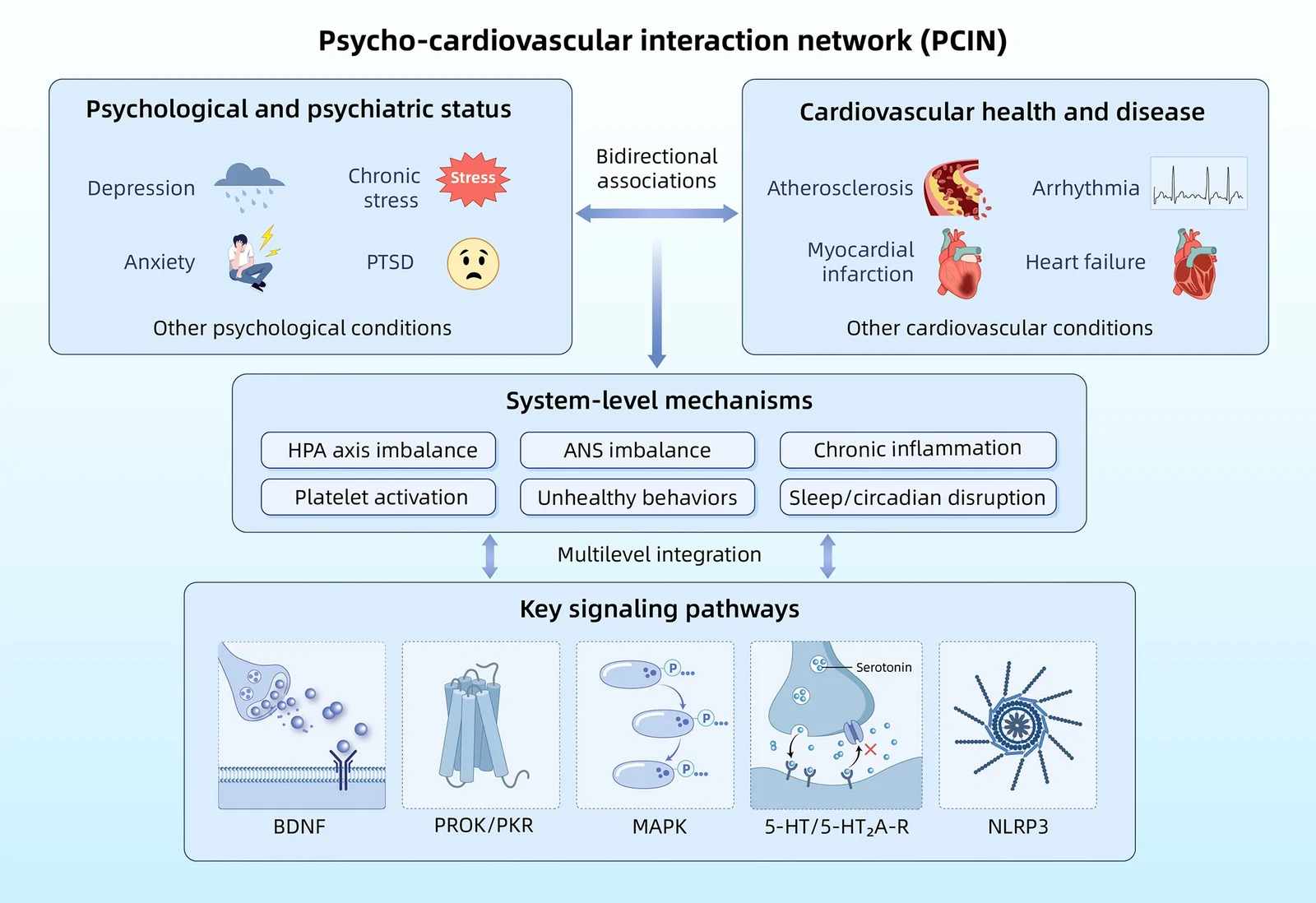
Overview of the psycho-cardiovascular interaction network (PCIN).

## Introduction

With the shift of modern medicine toward integration and precision, the impact of psychological and psychiatric factors on somatic diseases, especially cardiovascular disease (CVD), has received increasing attention. Psychosocial and psychiatric factors such as mood disorders, chronic stress, personality traits, and the social environment are closely associated with CVD onset, disease progression, and clinical outcomes [1–3].

Our understanding of the mind–body relationship has a long history. Philosophical concepts such as “the unity of body and spirit” assert that human health is the result of the dynamic balance of psychological, physiological, and environmental factors. Mental health status affects subjective well-being and behavioral decision-making, as well as influences physiological functions through multiple pathways such as the neuroendocrine system, immunomodulation, and autonomic nervous system (ANS). Recent cohort data further suggest that depression and anxiety are associated with adverse cardiovascular events, with stress-related neural activity, autonomic dysfunction, and systemic inflammation partly mediating these associations [4]. Psychological factors are no longer regarded solely as a reactive outcome of disease but are now recognized as potential etiological factors with important pathogenic and therapeutic implications [5]. In particular, psychological states such as depression, anxiety, and stress disorders can significantly increase the risk of CVDs, including hypertension, coronary heart disease (CHD), arrhythmia, and heart failure [6, 7].

**Figure 1:**
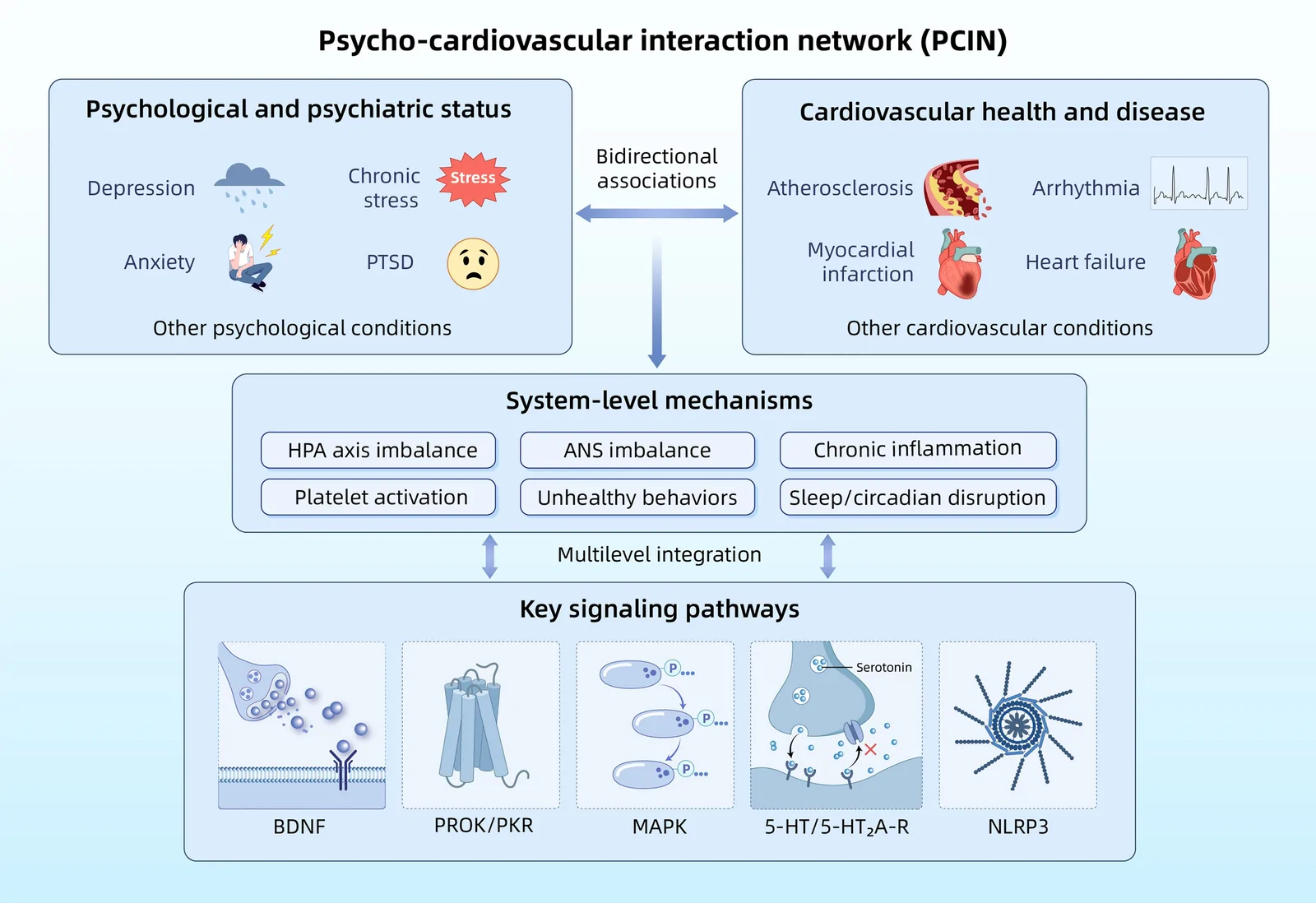
Overview of the psycho-cardiovascular interaction network (PCIN). PTSD: post-traumatic stress disorder; HPA: hypothalamic-pituitary-adrenal; ANS: autonomic nervous system; BDNF: brain-derived neurotrophic factor; PROK: prokineticin; PKR: prokineticin receptor; MAPK: mitogen-activated protein kinase; 5-HT: 5-hydroxytryptamine; NLRP3: NOD-like receptor protein 3.

In this context, elucidating the biological pathways, signal transduction mechanisms, and therapeutic targets involved in psycho-cardiovascular interactions and mental disorder–CVD comorbidity has become an important interdisciplinary research direction in basic and clinical medicine. However, most existing studies have focused on a single disease or mechanism and lack a systematic understanding of psycho-cardiovascular interactions. To address this limitation, we propose an integrative conceptual framework termed the psycho-cardiovascular interaction network (PCIN). The brain–heart axis broadly encompasses bidirectional communication between the nervous and cardiovascular systems [8]. Within this broader context, the PCIN specifically focuses on the links between psychological and psychiatric factors and cardiovascular health and disease, integrating epidemiological associations with system-level physiological mechanisms and molecular signaling pathways. Accordingly, the PCIN is not intended as a synonym for the brain–heart axis, but as an integrative psycho-cardiovascular framework in which brain–heart communication represents an important mechanistic component. An overview of the PCIN framework is provided in Fig. 1. On the basis of the PCIN framework, this article systematically reviews the biological basis of interactions between psychological factors and the cardiovascular system, and explores potential brain–heart axis-informed therapeutic strategies, with the aim of providing a basis for theoretical innovation and clinical translation in this field.

## Current state of epidemiology

CVDs are currently the leading cause of death worldwide [9]. Concurrently, the global burden of mental disorders has continued to rise. In 2021,  ~332.4 million people worldwide had depressive disorders [10], while  ~359.21 million had anxiety disorders [11]. The number of prevalent schizophrenia cases worldwide increased from 14.2 million to 23.6 million between 1990 and 2019 [12]. The absolute numbers of incident cases, prevalent cases, and DALYs for bipolar disorder also increased between 1990 and 2021 [13]. The burden of CVD is significantly higher in people with a mental disorder than in the general population [6, 7]. Data from the INTERHEART study demonstrated that psychosocial factors were strongly and independently associated with acute myocardial infarction [14]. The life expectancy of people with severe mental disorders is substantially shorter than that of the general population, which is mainly attributed to somatic diseases, with CVD being the leading cause of death [15]. The association is bidirectional, with patients with CVD also having an elevated risk of comorbid mental disorders [7, 16].

A meta-analysis of 26 studies involving 1 957 621 individuals found that depression was associated with incident myocardial infarction (HR 1.28, 95% CI 1.14–1.45), stroke (HR 1.13, 95% CI 1.00–1.28), and any CVD (HR 1.16, 95% CI 1.04–1.30), as well as cardiovascular mortality (HR 1.44, 95% CI 1.27–1.63) [17]. Another meta-analysis similarly found that depression was associated with higher cardiovascular mortality (HR 1.45, 95% CI 1.25–1.69) [18]. Depression and anxiety are also common among patients with CHD or heart failure [19]. A cohort study further showed that depression was associated with major adverse cardiac events (HR 1.24, 95% CI 1.14–1.34), with a stronger association when depression and anxiety co-occurred (HR 1.35, 95% CI 1.23–1.49) [4]. Patients with bipolar disorder and schizophrenia likewise have a substantial CVD burden and excess cardiovascular mortality [1, 20, 21].

Other psychological and psychiatric factors are likewise associated with CVD risk. Post-traumatic stress disorder (PTSD) has been associated with increased risks of CVD, myocardial infarction, and stroke [22]. Obsessive-compulsive disorder was found to be moderately associated with the risk of multiple types of CVDs [23]. Eating disorders, particularly anorexia nervosa, may be accompanied by serious cardiovascular complications. Common cardiac abnormalities in patients with anorexia nervosa include bradycardia, whereas QTc prolongation is variable and may be influenced by factors such as electrolyte disturbances and medications [24]. Early research reported that Type A personality was associated with an increased risk of CHD, which was independent of other risk factors [25, 26]. A recent meta-analysis identified a modest association between Type D personality and adverse cardiovascular outcomes in patients with CVD [27].

## Mechanisms regulating cardiovascular health within the PCIN

**Figure 2:**
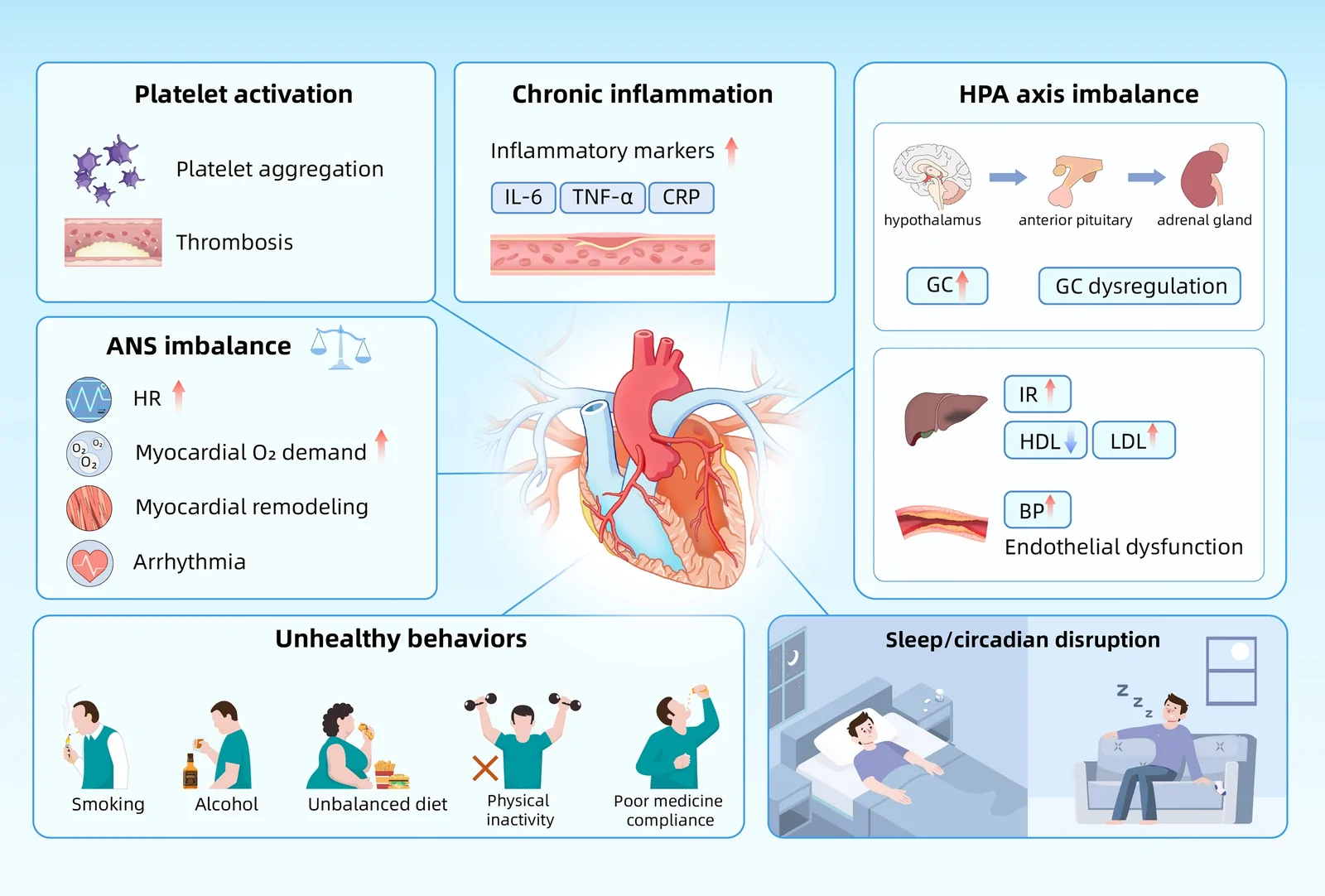
Mechanisms of cardiovascular health regulation within the PCIN. HPA: hypothalamic-pituitary-adrenal; IL-6: interleukin-6; TNF-α: tumor necrosis factor-α; CRP: C-reactive protein; GC: glucocorticoid; ANS: autonomic nervous system; IR: insulin resistance; HDL: high-density lipoprotein; LDL: low-density lipoprotein.

In this section, the potential mechanisms by which psychological factors influence cardiovascular health are summarized (Fig. 2). These mechanisms are closely interconnected within the PCIN and may jointly mediate the effects of psychological factors on cardiovascular function.

### Neuroendocrine axis imbalance

The hypothalamic-pituitary-adrenal (HPA) axis is a central pathway through which psychological stress affects the cardiovascular system. Under stress, the hypothalamus releases corticotropin-releasing hormone, which stimulates the pituitary gland to secrete adrenocorticotropic hormone. This ultimately leads to the release of glucocorticoids (GCs) from the adrenal gland. However, chronic psychological stress and psychiatric disorders, including depression, anxiety disorders, and PTSD, may lead to persistent dysregulation of HPA axis activity and feedback regulation, with alterations in GC secretion and circadian rhythmicity [28]. Depression and chronic stress have been associated with sustained elevations in cortisol in some populations, which are in turn associated with increased cardiovascular risk [29].

Excessive GC secretion is an important mechanistic basis for the promotion of CVD by psychological factors. Excess cortisol promotes insulin resistance, hyperglycemia, and dyslipidemia, thereby contributing to cardiometabolic and atherosclerotic risk [30]. With regard to vascular function, sustained hypercortisolism heightens the sensitivity of blood vessels to vasoconstrictors such as norepinephrine and angiotensin II. This causes sustained vasoconstriction and increased peripheral resistance, which leads to a higher risk of hypertension and increased left ventricular afterload [31, 32]. Hypercortisolism has been associated with endothelial dysfunction, increased arterial stiffness, and vascular remodeling, which may contribute to increased cardiovascular risk [31, 33].

### ANS imbalance

Psychological stress, anxiety, and depression can disrupt autonomic balance, leading to sympathetic overactivation and parasympathetic withdrawal, an important mechanism linking psychological factors to CVD [34]. Under stress, limbic–hypothalamic pathways increase sympathetic outflow and catecholamine release. The sympathetic nervous system enhances myocardial contractility and heart rate through β-adrenergic receptors. This increases myocardial oxygen consumption, which may induce myocardial ischemia or infarction. In addition, α-receptor-mediated peripheral vasoconstriction can trigger or exacerbate hypertension and increase left ventricular loading, thereby providing a pathological basis for structural cardiac remodeling [35]. Chronic sympathetic activation also promotes cardiomyocyte hypertrophy, collagen deposition, and structural remodeling, thereby increasing electrophysiological instability and the risk of arrhythmias and sudden death [36].

Heart rate variability (HRV) is an important biomarker of ANS status. Meta-analytic evidence indicates that HRV is reduced in major depressive disorder, generalized anxiety disorder, and panic disorder compared with healthy controls [37]. Reduced HRV has also been reported in patients with PTSD [38]. Lower HRV is associated with higher all-cause and cardiac mortality across healthy and clinical populations [39].

### Chronic inflammatory response activation

Patients with mental disorders such as depressive disorders, anxiety, and schizophrenia exhibit a low-grade chronic inflammatory state characterized by elevated levels of inflammatory markers, including C-reactive protein, interleukin-6 (IL-6), and tumor necrosis factor alpha (TNF-α) [40]. This persistent low-grade inflammation may contribute to cardiovascular injury by impairing endothelial nitric oxide-dependent vasodilation and promoting monocyte recruitment and foam-cell formation, thereby accelerating atherosclerotic plaque development [41]. IL-6 and TNF-α also regulate the migration and proliferation of vascular smooth muscle cells, thereby exacerbating plaque instability [42, 43]. In addition, chronic inflammation can cause interstitial fibrosis and cardiac remodeling. Moreover, IL-6 and TNF-α interfere with cardiac electrical activity through mechanisms such as sympathetic activation, disturbances in calcium homeostasis, and cardiac remodeling, thus increasing the risk of arrhythmias [44, 45].

### Platelet activation

Psychiatric and psychological factors modulate platelet function through the neuroendocrine-immune network, leading to abnormal enhancement of platelet activity and increasing the risk of thrombosis [46]. In depression, enhanced platelet activity may be linked to altered 5-hydroxytryptamine (5-HT) signaling, including changes in serotonin uptake and increased platelet reactivity to 5-HT [47]. Acute psychological stress leads to increased β-thromboglobulin levels in healthy volunteers and patients in stable condition after myocardial infarction [48]. Chronic psychological stress is also associated with platelet activation, with increased numbers of CD63-positive platelets and altered CD62P activation responses reported under chronic stress conditions [49]. Existing evidence suggests that patients with depression (with or without comorbid CHD) have elevated levels of coagulation and platelet activity markers, particularly platelet factor 4 and β-thromboglobulin [50]. Collectively, these alterations may contribute to increased thrombotic risk and cardiovascular events such as acute coronary syndrome.

### Unhealthy lifestyle behaviors

Psychological factors act indirectly on the cardiovascular system by influencing health-related behaviors [1]. Negative psychological states such as depression, anxiety, and chronic stress are associated with less healthy behavioral patterns, including smoking, excessive alcohol consumption, unhealthy diet, and physical inactivity [1, 51]. Smoking and excessive alcohol consumption damage the cardiovascular system through multiple mechanisms, including endothelial dysfunction, platelet activation and aggregation, lipid metabolism disorders, and chronic inflammatory activation. Patients with depression exhibit high-calorie, low-nutrient dietary patterns [52]. Diets rich in added sugars and saturated fats may contribute to atherosclerosis, endothelial dysfunction, and type 2 diabetes [53]. Physical inactivity is also a typical feature of depression [51]. Psychological factors also act indirectly on cardiovascular health by influencing treatment adherence. In patients with mental illness, treatment adherence is significantly lowered, and adverse psychological states may hinder them from seeking medical care, participating in rehabilitation, and complying with treatment regimens [1].

### Sleep/circadian rhythm disruption

As a fundamental physiological process for the maintenance of physical and mental health, sleep is closely related to psychological factors, jointly affecting cardiovascular function. Acute psychological stress promotes hyperarousal, delays sleep onset, and increases nocturnal awakenings [54]. Chronic stress-induced alterations in sleep architecture are more pronounced. Such changes manifest as an increase in the proportion of rapid eye movement sleep and a decrease in slow-wave sleep, thus affecting the restorative function of sleep and elevating cardiovascular health risks [55]. Recent umbrella evidence indicates that both short and long sleep durations are associated with adverse cardiometabolic and cardiovascular outcomes. Mendelian randomization analyses further suggest a potential causal relationship between short sleep and obesity, hypertension, and CHD, whereas comparable evidence for long sleep remains limited [56]. Insomnia has also been associated with multiple cardiovascular outcomes. Mendelian randomization evidence further supports potential causal associations with coronary artery disease, atrial fibrillation, heart failure, and hypertension [57]. Acute total sleep deprivation may alter autonomic and cortisol responses to stress, although the direction and magnitude of these responses appear heterogeneous rather than uniformly increased [58].

At the molecular level, these system-level processes involve multiple signaling pathways that contribute to psycho-cardiovascular interactions, as discussed in the following section.

## Key signaling pathways of the PCIN

Signaling within the PCIN is likely to involve a broad and interconnected molecular network. In this Review, we focus on five representative pathways: brain-derived neurotrophic factor (BDNF), prokineticin (PROK)/prokineticin receptor (PKR), mitogen-activated protein kinase (MAPK), 5-hydroxytryptamine (5-HT)/5-HT_2_A receptor (5-HT_2_A-R), and NOD-like receptor protein 3 (NLRP3) inflammasome signaling. These pathways span neurotrophic, peptidergic, kinase-mediated, monoaminergic, and inflammatory signaling, providing complementary perspectives on how neuropsychiatric and cardiovascular regulation may converge at the molecular level. Beyond these pathways, additional molecular mechanisms are likely to contribute to the complex signaling architecture of the PCIN.

### BDNF signaling pathway

**Figure 3:**
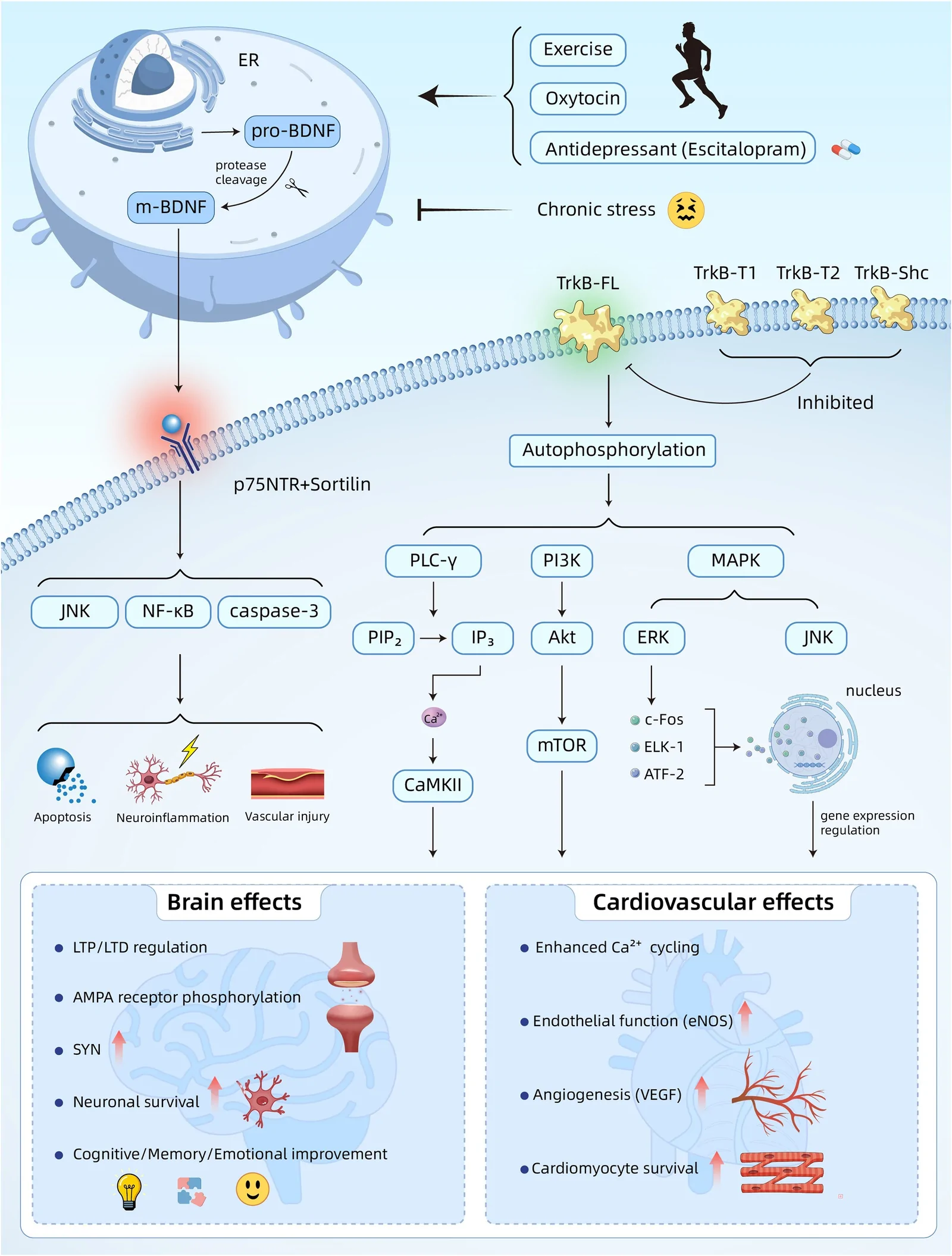
BDNF pathway in the PCIN and its dual effects on neuropsychiatric and cardiovascular systems. BDNF is secreted from the endoplasmic reticulum in the precursor form pro-BDNF, which is cleaved by proteolytic enzymes to produce m-BDNF. The expression of BDNF can be upregulated by stimuli such as exercise, oxytocin, and antidepressant drugs, and inhibited by chronic stress. m-BDNF preferentially binds to the full-length receptor TrkB-FL. This triggers autophosphorylation and the activation of multiple pathways such as PLC-γ/IP3/CaMKII, PI3K/Akt, and MAPK, thereby exerting protective effects in neuropsychiatric and cardiovascular systems. Truncated TrkB isoforms (TrkB-T1/T2/Shc) lack kinase activity and can thus inhibit TrkB-FL signaling. Upon binding to the p75NTR–sortilin complex, pro-BDNF activates the JNK, NF-κB, and caspase-3 pathways, leading to apoptosis, neuroinflammation, and vascular injury. ER: endoplasmic reticulum; BDNF: brain-derived neurotrophic factor; TrkB: tropomyosin receptor kinase B; p75NTR: p75 neurotrophin receptor; JNK: c-Jun N-terminal kinase; NF-κB: nuclear factor-kappa B; PLC-γ: phospholipase C-γ; PI3K: phosphatidylinositol 3-kinase; MAPK: mitogen-activated protein kinase; PIP2: phosphatidylinositol 4,5-bisphosphate; IP3: inositol 1,4,5-trisphosphate; Akt: protein kinase B; ERK: extracellular signal-regulated kinase; mTOR: mechanistic target of rapamycin; CaMKII: Calcium/calmodulin-dependent protein kinase II; LTP/LTD: long-term potentiation/long-term depression; AMPA: α-amino-3-hydroxy-5-methyl-4-isoxazolepropionic acid; SYN: synaptophysin; eNOS: endothelial nitric oxide synthase; VEGF: vascular endothelial growth factor.

Brain-derived neurotrophic factor (BDNF) is a core member of the neurotrophin family. BDNF is initially synthesized as the precursor pro-BDNF in the endoplasmic reticulum, which is subsequently cleaved by proteases into the mature form (m-BDNF). BDNF mediates its biological functions mainly through two types of receptors, namely the high-affinity tropomyosin receptor kinase B (TrkB) and the low-affinity p75 neurotrophin receptor (p75NTR). m-BDNF binds primarily to the TrkB receptor, whereas pro-BDNF preferentially binds to p75NTR. BDNF is widely distributed in the central nervous system (CNS) and plays an essential role in maintaining neuronal development, differentiation, survival, and synaptic plasticity [59]. BDNF is also abundantly expressed in cardiomyocytes, platelets, and damaged endothelial cells [60]. The trans-organ distribution of BDNF suggests that its signaling pathways play a crucial role in regulating the interactions between the CNS and cardiovascular system through the brain–heart axis (Fig. 3).

TrkB, a member of the tyrosine kinase receptor family, can be selectively spliced to generate full-length TrkB (TrkB-FL) and three truncated isoforms (TrkB-T1, TrkB-T2, and TrkB-Shc). The truncated receptors lack kinase activity and can inhibit downstream signaling pathway activation by interfering with the oligomerization and phosphorylation of TrkB-FL [61]. Binding of BDNF to TrkB-FL triggers its autophosphorylation and activates various signaling pathways, including the phospholipase C-γ/inositol 1,4,5-trisphosphate/calcium/calmodulin-dependent protein kinase II (PLC-γ/IP3/CaMKII), phosphoinositide 3-kinase/protein kinase B (PI3K/Akt), and MAPK pathways. Through this process, BDNF promotes neuronal growth, differentiation, survival, and synaptic plasticity, and participates in learning, memory, cognition, and emotion regulation [59]. The BDNF/TrkB signaling pathway can also regulate cardiomyocyte survival, vascular regeneration, and cardiac function [62]. In contrast, pro-BDNF preferentially binds to p75NTR and forms a trimeric complex with its co-receptor sortilin. This leads to the activation of the c-Jun N-terminal kinase (JNK) and nuclear factor kappa B (NF-κB) pathways, and promotes caspase-dependent apoptosis, neuroinflammation, and neuronal dysfunction [59, 63]. In the heart, the complex also induces cardiomyocyte injury and inflammatory responses through activation of the above pathways. Previous research has found that in myocardial hypoxia/reoxygenation injury, pro-BDNF promotes apoptosis of myocardial microvascular endothelial cells and participates in the pathogenesis of myocardial injury through activation of the p75NTR-sortilin signaling pathway and the downstream JNK and caspase-3 pathways [64].

In the CNS, the BDNF-TrkB signaling pathway regulates synapse formation through a variety of mechanisms, including the promotion of axonal and dendritic branching, formation of synaptic boutons, and stabilization of existing synapses. In the downstream signaling pathway, PLC-γ catalyzes the hydrolysis of phosphatidylinositol 4,5-bisphosphate (PIP2) to produce IP3 and diacylglycerol (DAG). Upon binding to its receptor, IP3 promotes the release of Ca^2+^, thereby activating CaMKII and regulating processes such as the cell cycle, cytoskeletal remodeling, and long-term potentiation (LTP)/long-term depression (LTD). CaMKII can also regulate its downstream targets, such as the α-amino-3-hydroxy-5-methyl-4-isoxazolepropionic acid (AMPA) receptor, glutamate ionotropic receptor AMPA type subunit 1 (GluA1), thereby promoting LTP expression [65]. BDNF also upregulates phosphorylation of the mechanistic target of rapamycin through activation of the PI3K/Akt pathway [59, 66]. This enhances synaptic transmission efficiency and the expression of synapse-associated proteins such as synaptophysin,  thereby promoting neuronal maturation and improve learning and memory abilities [67]. MAPK family members include extracellular signal-regulated kinase (ERK) and JNK, which are involved in the regulation of a variety of cellular processes [68, 69]. The ERK pathway can activate transcription factors (e.g. c-Fos, ELK-1, and ATF-2) to enter the nucleus, regulate gene expression, and promote CNS regeneration [70], whereas JNK mainly mediates inflammatory responses and apoptosis [71].

In the cardiovascular system, BDNF and its high-affinity receptor TrkB are expressed in the coronary endothelium and participate in capillary and endocardial endothelial formation [72]. In the peripheral vasculature, BDNF/TrkB promotes endothelial cell survival and hypoxia-induced angiogenesis by activating the Akt pathway, which contributes to the maintenance of vascular integrity. BDNF/TrkB also promotes vasodilation through the activation of endothelial nitric oxide synthase, thereby providing cardiovascular protective effects [73]. One study reported that TrkB-FL was primarily expressed in normal mouse myocardium, with BDNF enhancing Ca^2+^ cycling and elevating contractility through a CaMKII-dependent pathway, whereas truncated TrkB-T1 expression was elevated in a model of heart failure, which reduced the myocardial response to BDNF [74]. Exercise training can upregulate myocardial BDNF levels through activation of the PLC-γ/IP3/CaMKII and PI3K/Akt pathways, which contributes to the improvement of ejection fraction, stroke volume, and cardiac index after myocardial infarction [75]. BDNF also inhibits cardiomyocyte apoptosis, improves survival, and attenuates myocardial injury through the PI3K/Akt and MAPK pathways [76].

The onset and development of cardiocerebral comorbidities involve key mechanisms such as inflammation, oxidative stress, and HPA axis dysregulation. BDNF may play an important role in cardiocerebral comorbidities by regulating the aforementioned pathological processes. First, BDNF regulates inflammatory responses through multiple mechanisms. BDNF can inhibit glycogen synthase kinase 3β activity through the PLC-γ pathway and block inflammation-induced apoptosis [77]. BDNF also modulates oxidative stress. It possesses antioxidant properties and is capable of scavenging free radicals, activating the antioxidant enzyme system, and regulating the expression of vascular endothelial growth factor and myeloperoxidase, thus alleviating oxidative damage after acute myocardial infarction [76]. BDNF further enhances the ability of neurons to resist oxidative stress by upregulating antioxidant factors such as thioredoxin, and antidepressant drugs such as escitalopram may contribute to this antioxidant effect by upregulating BDNF levels [78, 79]. In addition, BDNF acts as an important hub for the regulation of HPA axis function. Chronic stress inhibits BDNF expression, and a decrease in BDNF level increases HPA axis activity, forming a vicious positive feedback loop [80]. Previous studies also found that oxytocin alleviated GC-induced depressive-like behavior by activating the CREB-BDNF pathway, whereas BDNF-deficient mice exhibited characteristics of excessive HPA axis activation. This further supports the existence of a dynamic inter-regulatory relationship between BDNF and the HPA axis. Such an imbalance may serve as an important mechanism for comorbidity between CVD and mental disorders [81]. Epidemiologic and genetic studies have also provided evidence for the role of BDNF in cardiocerebral comorbidities. Low circulating BDNF levels have been associated with adverse cardiovascular phenotypes [82], although evidence in CHD remains inconsistent [83]. In particular, the Val66Met polymorphism in the BDNF gene can affect its secretory efficiency and neuroplasticity, thereby altering an individual’s susceptibility to mental disorders and CVDs [84]. These findings support further investigation of BDNF as a potential biomarker and suggest that individualized intervention strategies can be developed based on genetic background.

### PROK/PKR signaling pathway

Prokineticins (PROKs) are a novel class of chemokine-like proteins with neuropeptide-like activity and pro-angiogenic hormone function [85]. The PROK family includes two members, namely prokineticin-1 (PROK1) and prokineticin-2 (PROK2). PROK1 and PROK2 perform their biological functions through interactions with two highly homologous G protein-coupled receptors: prokineticin receptor 1 (PROKR1, PKR1) and prokineticin receptor 2 (PROKR2, PKR2) [86]. Both PROK1 and PROK2 can bind and activate PKR1 (393 amino acids) and PKR2 (384 amino acids), with PROK2 having a higher affinity for PKR1 [87]. PROK and PKR exist in a wide variety of organs and tissues, including the CNS, heart, gastrointestinal tract, adrenal gland, bone marrow, and blood [88].

**Figure 4:**
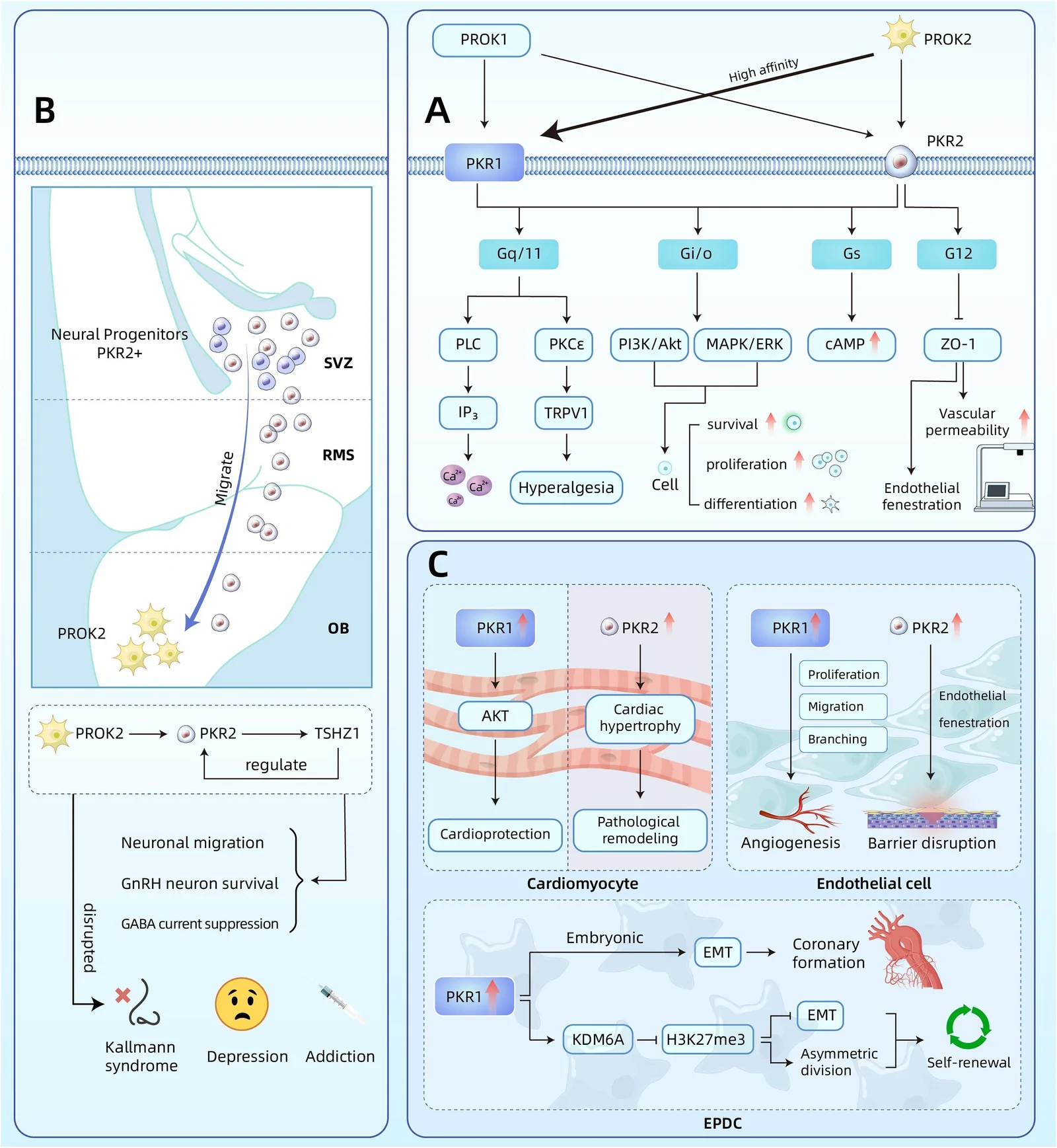
PROK/PKR pathway in PCIN. A. PROK/PKR signaling pathway; B. The effect of PROK2/PKR2 in the neuropsychiatric system; C. The effect of PROK2/PKR2 in the cardiovascular system. (A) PROK1 and PROK2 perform their biological functions and mediate multiple downstream signaling pathways by interacting with the PKR1 and PKR2 receptors, with PROK2 having a higher affinity for PKR1. PKR1/2 can activate the PLC-IP3-Ca^2+^ pathway and induce PKCε translocation via Gq/11, which enhances TRPV1 activity and leads to hyperalgesia. PKR1/2 can also activate the PI3K/Akt and MAPK/ERK pathways via Gi/o, thereby promoting cell survival, proliferation, and differentiation, and promote cAMP accumulation via Gs. PKR2 also inhibits ZO-1 protein expression via G12, which increases vascular permeability and induces endothelial cell fenestration.(B)PROK2 can upregulate TSHZ1 via PKR2, and regulate neuronal migration, GnRH neuron survival and GABA current inhibition. In the adult mouse brain, PKR2+ neural progenitor cells migrate from the SVZ to the OB along the RMS, and PROK2 acts as a chemokine to induce their migration. Abnormalities in their function have been associated with Kallmann syndrome, depression, and addiction. (C) In cardiomyocytes, PKR1 exerts cardioprotective effects through the Akt pathway, whereas PKR2 induces myocardial hypertrophy and pathological remodeling. In endothelial cells, PKR1 promotes cell proliferation, migration, branching and angiogenesis, whereas PKR2 leads to endothelial cell fenestration and disruption of barrier function. In EPDC, PKR1 regulates embryonic EMT and participates in coronary artery formation. PKR1 also promotes asymmetric division and self-renewal by removing the H3K27me3 repressive marker via KDM6A. PROK: prokineticin; PKR: prokineticin receptor; PLC: phospholipase C-γ; IP3: inositol 1,4,5-trisphosphate; PKCε: protein kinase Cε; TRPV1: transient receptor potential vanilloid 1; PI3K: phosphoinositide 3-kinase; MAPK: mitogen-activated protein kinase; ERK: extracellular signal-regulated kinase; SVZ: subventricular zone; RMS: rostral migratory stream; OB: olfactory bulb; TSHZ1: teashirt zinc finger family member 1; GnRH: gonadotropin-releasing hormone; GABA: gamma-aminobutyric acid; EPDC: epicardium-derived cell; EMT: epithelial-mesenchymal transition; KDM6A: lysine demethylase 6A; H3K27me3: histone H3 lysine 27 trimethylation.

The activated PROK/PKR pathway can mediate multiple downstream signaling pathways by coupling to multiple G proteins [89]. In particular, the Gq/11-mediated pathway promotes IP3 synthesis and intracellular Ca^2+^ mobilization through activation of PLC [86] and induces the translocation of protein kinase Cε (PKCε) to the plasma membrane [90]. This enhances transient receptor potential vanilloid 1 (TRPV1) channel activity, thereby exacerbating the PKR2-mediated hyperalgesic response [91]. PKR2 also couples with G12 in endothelial cells and inhibits expression of the tight junction protein ZO-1, thus inducing endothelial cell fenestration and increasing vascular permeability [92]. In addition, PKRs can activate the MAPK/ERK pathway via Gi/o proteins [86] and promote endothelial cell differentiation, proliferation, and migration via the PI3K/Akt pathway [93]. Activation of PKR1 and PKR2 also promotes cAMP accumulation via Gs proteins, which regulates cellular functions such as myocardial contraction [94]. These signaling mechanisms form the molecular basis for the functions of the PROK/PKR system in various physiological and pathological processes. In particular, they exert key regulatory effects in cardiovascular and neurological diseases, and may play an important role in the regulation of the brain–heart axis [88] (Fig. 4).

PKR1 and PKR2 are widely expressed in the cardiovascular system, including cardiomyocytes, epicardial progenitor cells (EPDCs), endothelial cells, and fibroblasts [95]. Through mechanisms mediated by multiple cell types, they play key roles in cardiac development, myocardial protection, and angiogenesis. In cardiomyocytes, the activation or overexpression of PKR1 enhances cell survival through the Akt signaling pathway, thereby exerting protective effects against hypoxic injury [92, 96]. Previous research reported that PKR1-overexpressing transgenic mice showed a significant increase in the number of EPDCs, density of capillaries, and number of coronary arteries. In addition, PKR1 signaling upregulated the expression of its own ligand, PROK2, which acts as a cardiac secretory factor that induces differentiation of EPDCs into endothelial and smooth muscle cells and promotes angiogenesis [97]. In contrast, the activation or overexpression of PKR2 in cardiomyocytes induces a hypertrophic response and impairs endothelial function, suggesting that the two PROK receptors have distinct biological functions in cardiomyocytes [98]. In coronary artery endothelial cells, the activation or overexpression of PKR1 can stimulate cell proliferation, migration, and branching, thereby promoting angiogenesis, partly through MAPK and Akt signaling [99, 100]. By contrast, PKR2 activation in endothelial cells does not promote angiogenesis [99, 101]. PKR2 signaling induces a fenestrated endothelial cell phenotype, resulting in the loss of the intercellular junction protein ZO-1 at tight junctions and disorganization of the endothelial structure [101]. PKR1 and PKR2 can be co-expressed in cardiac capillary endothelial cells, with the former predominantly expressed under physiological conditions and PKR2 expression enhanced in response to pathological stimuli [100]. Therefore, the role of PKR2-specific antagonists in the regulation of endothelial function remains to be further investigated.

PKR1 is also capable of regulating the fate and epithelial-mesenchymal transition (EMT) of EPDCs. In the embryonic stage, PROK2 promotes the EMT of EPDCs through activation of PKR1, which then migrate and participate in the formation of the coronary vascular network [102]. Human EPDCs derived from the atrial appendage can spontaneously undergo EMT *in vitro*, but their differentiation potential is limited. PKR1 suppressed EMT and induced asymmetric division by activating lysine demethylase 6A to inhibit the repressive histone H3 lysine 27 trimethylation marks. This led to self-renewal and formation of vascular and epithelial/endothelial precursors, which had angiogenic potential and were capable of differentiating into endothelial and smooth muscle cells [103].

The PROK/PKR signaling pathway is of vital significance in olfactory bulb (OB) development and the survival and migration of gonadotropin-releasing hormone (GnRH) neurons. In the adult mouse brain, PKR2 is widely expressed in neurogenically active regions, such as the subventricular zone (SVZ), rostral migratory stream (RMS), and the ventricular and paraventricular layers of the olfactory ventricle (OV) [104]. PROK2 is expressed in the granular and peribulbar layers of the OB and can act as a chemokine for SVZ-derived neural progenitor cells, inducing their migration [105]. Studies using genetic mouse models further confirmed the presence of PROK2+ cells in the medial part of the RMS, whereas PKR2 is expressed in specific neuronal cells in the OB, such as the granule cells and tufted cells [105]. Loss-of-function models have further revealed the role of PROK/PKR in neurodevelopment. PROK2-deficient mice (PROK2^−/−^) exhibited a reduced OB volume and a large accumulation of neural progenitor cells in the RMS region, suggesting the impairment of neural migration [106]. Similarly, PKR2-deficient mice (PKR2^−/−^) showed hypoplasia and structural disorganization in the OB and an absence of GnRH neurons in the hypothalamus [107]. These phenotypes are similar to the clinical features of Kallmann syndrome, which is characterized by gonadotropin deficiency and anosmia [108], and several mutations in the PROK2 and PKR2 genes have also been reported to be associated with this disease [109]. The development of inhibitory interneurons in the OB (e.g. GABAergic neurons) is dependent on the transcription factor TSHZ1 (teashirt zinc finger family member 1) downstream of PKR2. TSHZ1 directly binds to and regulates PKR2 expression, and its conditional knockout results in OB hypoplasia and significant olfactory deficits, with the phenotype being similar to that of PKR2-deficient mice [110]. Tshz1 mutant mice exhibited a loss of amygdala intercalated cells accompanied by abnormalities in fear responses, depressive behavior, and social interaction. PROK2 also inhibits GABA-mediated current conduction in sensory neurons [111]. PROK2 or PKR2 deficiency impairs neuroblast migration along the SVZ-RMS-OB axis and reduces GABAergic interneurons in the OB, suggesting that PROK2/PKR2 signaling mainly regulates the migration and survival of GnRH neurons and the migration of OB interneurons [104, 105]. PKR2 has also been found to be associated with a variety of mental disorders. For instance, its aberrant expression has been associated with major depressive disorder [112] and the risk of methamphetamine dependence [113], indicating that this pathway may play a role in emotion regulation and addiction mechanisms.

In short, the PROK/PKR signaling pathway regulates neurogenesis and angiogenesis during neural and cardiac development, and plays critical roles in protection and repair after cardiocerebral injury. Activation of pro-survival signaling in myocardial and neuronal cells through autocrine/paracrine modes may provide a new strategy for the treatment of diseases related to the brain–heart axis. Recent evidence further highlights the distinct roles of PKR1 and PKR2 and the therapeutic potential of receptor-targeted ligands, although support remains largely preclinical [114]. The in-depth analysis of the mechanisms of PROK/PKR signaling in brain–heart interactions in future research will offer a new theoretical basis and intervention targets for psycho-cardiovascular association.

### MAPK signaling pathway

The MAPK signaling pathway is a highly conserved intracellular signaling network responsible for transmitting a variety of extracellular stimuli from the membrane to the nucleus. The canonical MAPK signaling cascade consists of three tiers of kinases that activate each other sequentially: MAPK kinase kinase (MAPKKK, also known as MKKK), MAPK kinase (MAPKK, also known as MKK), and terminal MAPK [115]. In the human genome, the MAPK family consists of multiple subfamily members usually divided into four major subgroups: ERK1/2, p38 MAPK (including isoforms such as p38α/β), JNK, and ERK5 [116]. These MAPK branches integrate growth- and stress-related signals and participate in processes including proliferation, differentiation, inflammation, apoptosis, and tissue remodeling [69].

Within the CNS, the MAPK pathway mediates neuronal and glial responses to a variety of stimuli, including neurotransmitters, growth factors, stress hormones, and inflammatory mediators. Ultimately, the activated MAPK translocates to the nucleus to regulate gene expression, thereby affecting neuronal plasticity and survival [117]. In the brain, the ERK1/2 pathway is closely related to synaptic plasticity and memory formation. ERK/MAPK activity regulation may determine the switch between memory consolidation and extinction, and can be regarded as a novel target for the treatment of anxiety and PTSD. Accordingly, aberrant ERK signaling may lead to corresponding cognitive or emotional dysfunction [118].

The JNK and p38 pathways are more commonly associated with stress- and inflammation-related neurological changes. JNK is a classical stress kinase, and a large number of studies have indicated that JNK signaling is overactive in mental disorders such as depression. Multiple pathological factors of depression (e.g. neuroinflammation, excitotoxicity, oxidative stress, cerebral ischemia, and psychological stress) can significantly upregulate JNK activity [119]. In addition, the use of JNK inhibitors significantly attenuated inflammation-induced depressive-like responses, and the efficacy of certain antidepressants has been associated with the inhibition of JNK activity. These findings collectively support the notion that JNK is an important regulatory pathway in the onset of depression [120, 121]. p38 MAPK also serves a crucial role in the central stress response. Chronic stress induces a decrease in serotonergic function through activation of p38α MAPK in midbrain 5-HT neurons. This leads to insufficient serotonin secretion, which promotes the occurrence of depressive-like behaviors. Selective knockout of p38α in 5-HT neurons results in mice exhibiting a stress-resistant behavioral phenotype in response to stress [122, 123]. p38 pathway-mediated inflammatory responses in astrocytes and microglia are also believed to be involved in the effects of psychological stress on the brain: p38α activation in microglia induces the release of large quantities of pro-inflammatory cytokines that drive stress-related neuroinflammatory responses [124]. Such neuroinflammation is regarded as an important link in the pathology of depression and PTSD, and is characterized by the interference of downstream receptor function by elevated levels of inflammatory factors through signaling pathways (including p38 MAPK). For instance, excessive levels of TNF-α can influence the activity and expression of the 5-HT transporter (SERT) via the MAPK pathway. This leads to increased serotonin reuptake, thereby aggravating neurotransmitter imbalance [125]. In summary, various branches of the MAPK pathway participate in the onset of neurological and psychiatric diseases such as depression, anxiety, and PTSD by affecting neuronal plasticity, neurogenesis, and inflammatory mediator production.

In the cardiovascular system, the MAPK signaling pathway is a key pathway that mediates the responses of cardiomyocytes, fibroblasts, and vascular cells to various physicochemical stimuli and injuries. When the myocardium is subjected to mechanical stress, ischemia and hypoxia, or inflammatory factors, MAPK pathways such as ERK, JNK, and p38 are rapidly activated. These pathways are collectively involved in the processes of myocardial hypertrophy, cell death, fibrosis, and vascular remodeling, although different specific functions are performed by different subgroups [126]. In particular, ERK1/2 and JNK1/2 overactivation can synergistically stimulate cardiac fibroblast proliferation and promote accelerated progression of myocardial fibrosis [127]. Inhibition of these pathways (including p38, ERK, and JNK) can aid in attenuating myocardial injury and fibrosis caused by oxidative stress, which is expected to delay the deterioration of cardiac function [128].

The MAPK pathway is also involved in regulating vascular endothelial function and vascular remodeling. Inflammatory factors or oxidative stress can activate p38 and JNK in vascular endothelial cells and smooth muscle cells, causing upregulation of the expression of adhesion molecules and cytokines. This facilitates the adhesion and migration of inflammatory cells into the vascular wall, thus exacerbating endothelial dysfunction and vascular inflammatory responses [129]. The deleterious effects of p38 and JNK signaling have also been observed in myocardial ischemia-reperfusion injury: ischemic stress can overactivate p38α, which induces undesirable changes such as cardiomyocyte apoptosis and the release of inflammatory factors. The timely inhibition of p38 activity has been found to reduce myocardial infarct size and improve cardiac function [130].

**Figure 5:**
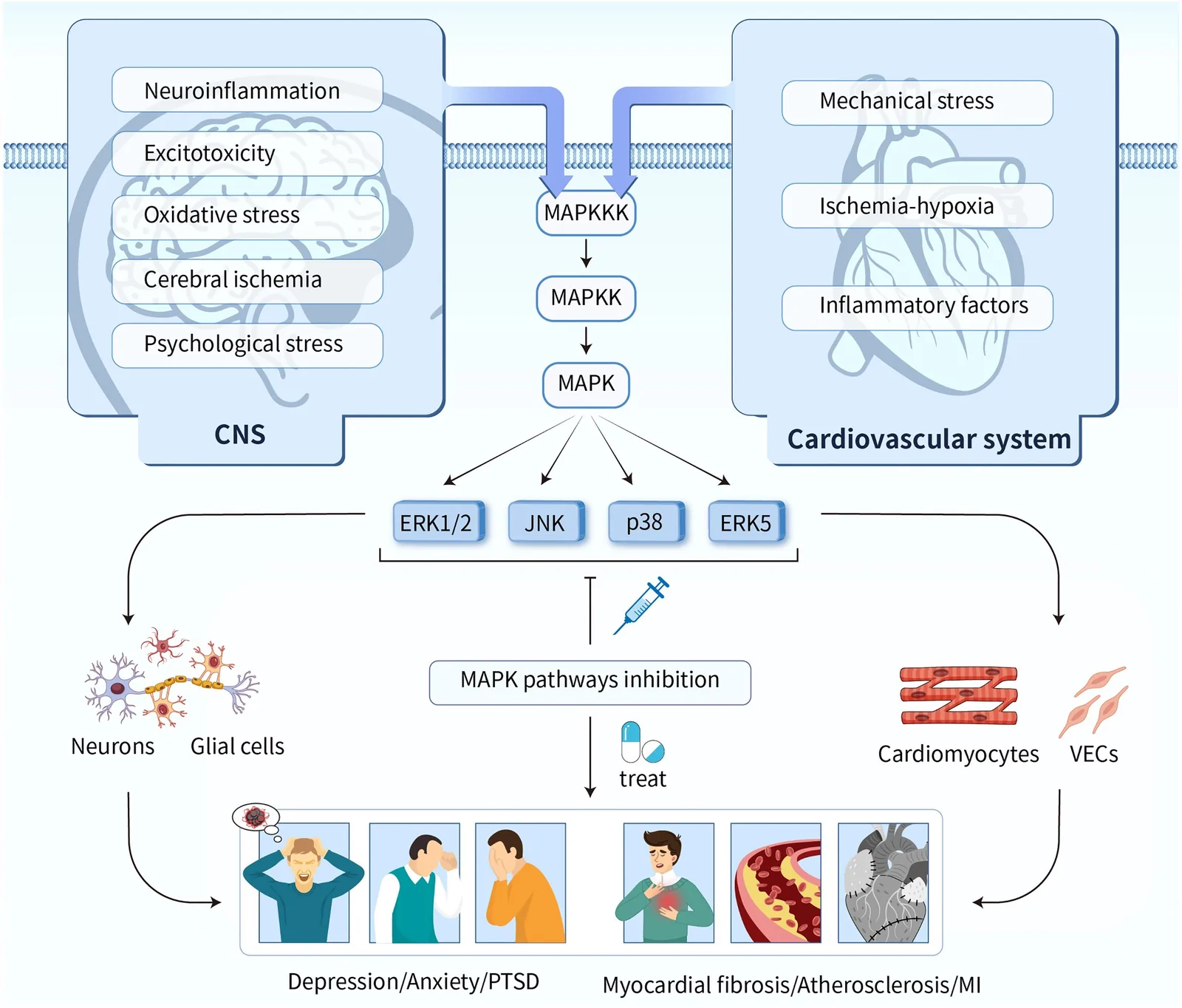
MAPK pathway in PCIN and its dual effects on neuropsychiatric and cardiovascular systems. The MAPK pathway mediates pathophysiological changes in the CNS and cardiovascular system. In the CNS, stimuli such as neuroinflammation, excitotoxicity, oxidative stress, cerebral ischemia, and psychological stress activate the MAPK cascade; in the cardiovascular system, the pathway is triggered by mechanical stress, ischemia-hypoxia, and inflammatory factors. These stimuli induce signals that are propagated through a three-tiered kinase cascade and ultimately activate members of the four major MAPK subgroups (ERK1/2, JNK, p38, and ERK5). Activated MAPK signaling can act on neurons, glial cells, cardiomyocytes, and VECs, leading to depression, anxiety, and PTSD in the neuropsychiatric system, and myocardial fibrosis, atherosclerosis, and myocardial infarction in the cardiovascular system. MAPK pathway could be potential therapeutic targets. CNS: central nervous system; MAPK: mitogen-activated protein kinase; MAPKK: MAPK kinase; MAPKKK: MAPK kinase kinase; ERK: extracellular signal-regulated kinase; JNK: c-Jun N-terminal kinase; VECs: vascular endothelial cells; PTSD: post-traumatic stress disorder; MI: myocardial infarction.

Systemic low-grade inflammatory responses are also an important factor in the comorbidity between mental disorders and CVD. Their systemic effects can affect target organs through the MAPK pathway; e.g. the excessive release of stress hormones and catecholamines can activate the ERK1/2 pathway within cardiomyocytes through the β-adrenergic receptor [131, 132]. Animal studies have reported that p44/42 ERK was transiently activated in the heart of rats subjected to acute restraint stress, and this activation could be blocked by the preadministration of β1 adrenergic blockers [133]. This phenomenon suggests that psychological stress can directly trigger myocardial MAPK signaling, causing alterations in cardiac electrophysiology and contractile function. Inflammatory factors can also aggravate endothelial dysfunction and local inflammatory infiltration by activating p38/JNK signaling in endothelial and immune cells, thereby promoting atherosclerotic plaque formation [134]. Certain natural polyphenols provide dual antidepressant and cardioprotective effects. The pertinent mechanisms involve the inhibition of MAPK-mediated oxidative stress and inflammatory pathways, thereby concurrently alleviating depressive-like behaviors and myocardial injury [135, 136]. Thus, persistent low-grade inflammation and imbalanced stress responses are common features of both “cardiogenic” psychological problems (e.g. secondary depression and anxiety in patients with heart disease) and “neurogenic” cardiac problems (e.g. cardiovascular complications in patients with mental disorders). The MAPK signaling pathway is integral to all of these processes and is essential for bidirectional brain–heart regulation (Fig. 5).

### 5-HT/5-HT_2_A-R pathway

5-HT is a biogenic amine with both neurotransmitter and peripheral hormone functions and is widely involved in mood regulation, cognition, and sleep, as well as a variety of physiological processes such as vascular tone, platelet aggregation, and inflammation [137]. Mental disorders may mediate cardiovascular abnormalities through the 5-HT/5-HT_2_A receptor (5-HT_2_A-R) signaling pathway, one of the important mechanisms of the brain–heart axis interactions between the CNS and the cardiovascular system. 5-HT_2_A-R belongs to the G protein-coupled receptor family and mainly activates the PLC signaling pathway through the Gαq protein. After PLC activation, PLC hydrolyzes PIP2 on the cell membrane, producing two important second messengers, IP3 and DAG. IP3 binds to IP3 receptors on the endoplasmic reticulum, thus triggering the opening of the calcium-ion channel. This causes a massive release of Ca^2+^ stored in the endoplasmic reticulum into the cytoplasm, leading to a drastic increase in intracellular Ca^2+^ concentration [138]. Meanwhile, DAG activates PKC on the cell membrane, thereby regulating a variety of cell biological functions. The receptor is widely expressed in tissues such as the cerebral cortex, platelets, vascular smooth muscle cells, and the heart, and could potentially function as a key molecule linking the mental and cardiovascular systems [139, 140].

5-HT_2_A-R plays a key role in the pathogenesis of many mental disorders, including schizophrenia [141], obsessive-compulsive disorder [142], anxiety disorders [143], eating disorders [144], and attention-deficit hyperactivity disorder [145]. Functional abnormalities are often present in 5-HT_2_A-R-mediated signaling pathways in the prefrontal cortex (PFC) of patients with these disorders [143, 146]. In the PFC, 5-HT_2_A-R can inhibit membrane Ca_v_1.2 L-type Ca^2+^ currents via the Gαq-mediated PLCβ-IP3-calcineurin signaling pathway, whereas the PKC pathway is not involved in this modulation process [147]. After PLC activation, IP3 binds to endoplasmic reticulum receptors to promote calcium release. The released Ca^2+^ activates calcineurin, leading to the inhibition of Ca_v_1.2 L-type Ca^2+^ currents by dephosphorylation [148], thereby affecting neuronal excitability and synaptic transmission.

A study found that excitatory inward currents were generated in  ~40% of layer V PFC neurons after 5-HT_2_A-R activation in adult rats with a history of early stress [149]. This animal model demonstrated that early stress can lead to a marked enhancement of 5-HT_2_A-R function in the PFC [149], thus providing a potential neural mechanism for explaining the increased susceptibility of individuals to anxiety and depression in adulthood. In a postmortem study, measurement of GTPγS binding revealed that 5-HT_2_A-R function was enhanced in the PFC of patients with bipolar affective disorder [150]. Another study found that the administration of MDL 11,939, a 5-HT_2_A-R antagonist, alleviated stress-related behavioral abnormalities in a rat model of learned helplessness stress [151].

In the cardiovascular system, 5-HT can similarly regulate vascular smooth muscle tone, heart rate, platelet function, and inflammatory responses through its multiple receptor subtypes [139]. 5-HT_2_A-R activation induces vasoconstriction, and 5-HT_2_A-R-activated vascular smooth muscle cells can trigger the release of massive amounts of Ca^2+^ from the sarcoplasmic reticulum through the Gq-PLC-IP3 signaling pathway. Intracellular free Ca^2+^ binds to calmodulin (CaM) to form a Ca^2+^-CaM complex, which directly activates myosin light chain kinase. This leads to myosin light chain phosphorylation and actin-myosin cross-bridge cycling, resulting in vascular smooth muscle contractions [152]. Src kinase (Src) and PI3K are also activated, which in turn induces epidermal growth factor receptor (EGFR) transactivation. The Gβγ subunit of 5-HT_2_A-R also synergistically activates Src/PI3K. EGFR transactivation in turn activates the downstream ERK1/2 and PI3K/Akt signaling pathways. Consequently, this mediates vasoconstriction in the short term and drives cell proliferation in the long term, leading to involvement in pathological processes [153]. 5-HT_2_A-R also enhances platelet activation and aggregation via the Gq-PLC pathway. PKC is jointly activated by Ca^2+^ mobilization generated by the Gq-PLC pathway and DAG. Upon activation, PKC participates in inside-out signaling and drives the switch of the conformation of integrin αIIbβ3 on the platelet membrane surface from a “bent” inactive state to a “stretched” active state, thereby exposing binding sites for fibrinogen. Elevated Ca^2+^ activates cytosolic phospholipase A_2_, which catalyzes the release of arachidonic acid from membrane phospholipids and generates thromboxane A_2_, thereby further amplifying the platelet aggregation response [154, 155]. 5-HT_2_A-R activation is involved in atherosclerosis and thrombosis. In macrophages, upon the activation of 5-HT_2_A-R by oxidized low-density lipoprotein (LDL) and fatty acids, PKCε is activated via the Gq-PLC-DAG signaling pathway, which in turn upregulates glycerol-3-phosphate acyltransferase 1 expression to promote triglyceride synthesis, and activates CD36 receptors to increase oxidized LDL uptake. In vascular endothelial cells, lipid stimulation increases 5-HT synthesis while enhancing monoamine oxidase A activity, which catalyzes 5-HT degradation. This leads to the generation of a large number of mitochondrial reactive oxygen species, activation of the NF-κB signaling pathway, and the release of inflammatory factors such as monocyte chemoattractant protein-1, TNF-α, and IL-1β, thus promoting monocyte adhesion and infiltration [156]. These findings highlight the pathological significance of 5-HT_2_A-R-related pathways in CVD.

Patients with mental disorders often exhibit alterations such as decreased 5-HT concentration in peripheral blood and upregulated 5-HT_2_A-R expression in platelets, suggesting that abnormalities in the 5-HT/5-HT_2_A-R signaling pathway associated with psychiatric factors are not confined to the CNS and may spread to peripheral organs, particularly the cardiovascular system. Disturbances in the 5-HT system induced by mental disorders affect neuropsychiatric function, as well as significantly increase the risk of cardiovascular events through the mediation of abnormal vascular function, enhanced inflammatory responses, and imbalanced neuroendocrine regulation by peripheral 5-HT_2_A-R [157, 158]. Chronic stress elevates 5-HT_2_A-R expression levels, and activation of the receptor inhibits parasympathetic synaptic transmission and promotes the development of stress-related disorders (e.g. depression and anxiety disorders) and CVD [159]. The 5-HT_2_A-R system can mediate brain–heart regulation through interrelated mechanisms such as autonomic pathways and inflammatory mediators [160]. In the CNS, 5-HT_2_A-R is highly expressed in the medullary cardiovascular regulatory centers, including the nucleus tractus solitarius, the dorsal nucleus of the vagus nerve, and the rostral ventrolateral medulla. Therefore, these brain regions may be involved in the regulation of cardiovascular function by the ANS [161]. However, the immunomodulatory effects of 5-HT_2_A-R signaling appear to be cell- and context-dependent. In other experimental settings, 5-HT_2_A-R activation exhibits potent anti-inflammatory effects, inhibiting TNF-α-induced expression of various pro-inflammatory genes and preventing increases in circulating IL-6 levels [162].

**Figure 6:**
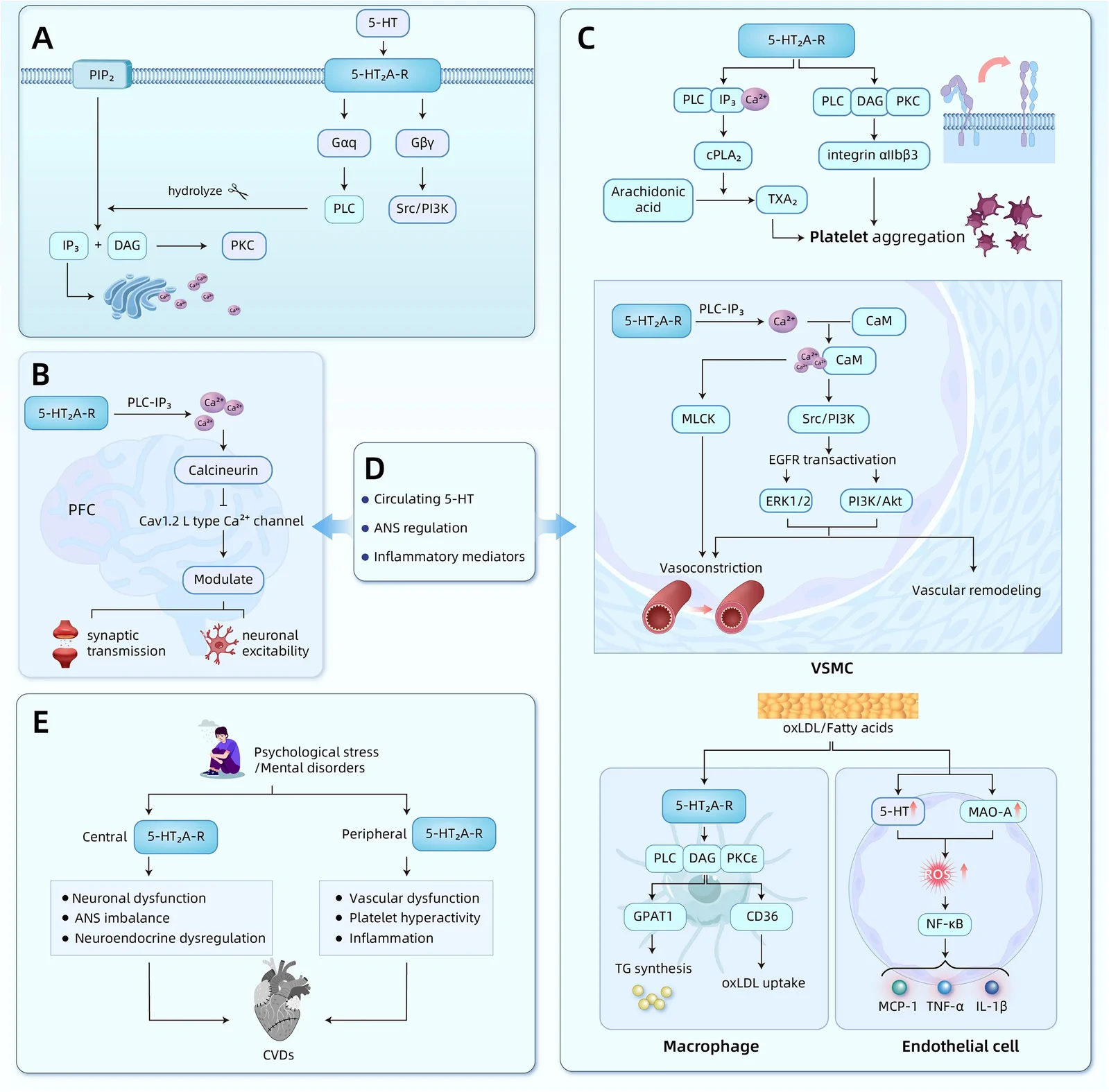
5-HT/5-HT_2_A-R pathway in PCIN. A. 5-HT_2_A-R signaling pathway; B. The effect of 5-HT_2_A-R in PFC; C. The effect of 5-HT_2_A-R in the cardiovascular system; D. The mechanisms of 5-HT signaling linking neuropsychiatric and cardiovascular systems; E. Psychologically mediated cardiovascular dysfunction through central and peripheral 5-HT_2_A-R.(A) Binding of 5-HT to 5-HT_2_A-R results in activation of PLC, which hydrolyzes PIP2 to generate IP3 and DAG. IP3 promotes calcium release, whereas DAG activates PKC. (B) In the PFC, 5-HT_2_A-R activates calmodulin phosphatase via PLC-IP3-Ca^2+^ signaling, inhibits Ca_v_1.2 L-type calcium channels, and regulates synaptic transmission and neuronal excitability. (C) In platelets, activated 5-HT_2_A-R promotes cPLA_2_ activation via PLC-IP3-Ca^2+^ to generate TXA_2_, whereas activation of integrin αIIbβ3 via PLC-DAG-PKC mediates platelet aggregation. In VSMC, 5-HT_2_A-R activation leads to the release of Ca^2+^. This leads to the formation of the Ca^2+^-CaM complex, which activates MLCK and Src/PI3K and mediates vasoconstriction and remodeling. oxLDL/fatty acids activate macrophage 5-HT_2_A-R, which upregulates GPAT1 and CD36 via the PLC-DAG-PKCε pathway, thereby promoting triglyceride synthesis and oxLDL uptake. In endothelial cells, 5-HT generates ROS through MAO-A degradation and activates NF-κB to release inflammatory factors. (D) The mechanisms of 5-HT signaling linking neuropsychiatric and cardiovascular systems. (E) Psychological stress and mental disorders affect cardiovascular function through central and peripheral 5-HT_2_A-R, leading to CVD. 5-HT: 5-hydroxytryptamine; 5-HT_2_A-R: 5-HT_2_A receptor; PIP2: phosphatidylinositol 4,5-bisphosphate; PLC: phospholipase C; IP3: inositol 1,4,5-trisphosphate; DAG: diacylglycerol; PI3K: phosphoinositide 3-kinase; PKC: protein kinase C; cPLA_2_: cytosolic phospholipase A_2_; TXA_2_: thromboxane A_2_; VSMC: vascular smooth muscle cell; CaM: calmodulin; MLCK: myosin light chain kinase; EGFR: epidermal growth factor receptor; ERK: extracellular signal-regulated kinase; Akt: protein kinase B; oxLDL: oxidized low-density lipoprotein; GPAT1: glycerol-3-phosphate acyltransferase 1; TG: triglyceride; MAO-A: monoamine oxidase A; ROS: reactive oxygen species; NF-κB: nuclear factor kappa B; MCP-1: monocyte chemoattractant protein-1; TNF-α: tumor necrosis factor-α; IL-1β: interleukin-1β; ANS: autonomic nervous system; CVD: cardiovascular disease.

In a mouse model of depression induced by chronic unpredictable mild stress, decreased brain and plasma 5-HT levels mediated depression-associated myocardial injury and cardiomyocyte apoptosis. Selective 5-HT reuptake inhibitors (SSRIs) such as sertraline significantly reduced cardiomyocyte apoptosis. A mouse model of transverse aortic constriction confirmed that M100907 inhibited Gq-PLC pathway-mediated calcium signaling through selective blockade of 5-HT_2_A-R. This, in turn, blocked the downstream CaMKII/histone deacetylase 4 (HDAC4) phosphorylation cascade, thereby deterring pathological hypertrophic gene transcription and improving cardiac remodeling [163]. Collectively, 5-HT_2_A-R plays an important role in the bidirectional regulation of the brain–heart axis by integrating networks of nerves, signaling molecules, and inflammatory mediators (Fig. 6). Recent translational evidence supports a role for peripheral serotonin in platelet activation, vascular tone regulation, endothelial dysfunction, and vascular remodeling. However, its clinical utility as a biomarker remains investigational because standardized measurement methods and large prospective validation studies are still lacking [164].

### NLRP3 inflammasome pathway

Previous research has suggested that mental disorders, particularly depression, may mediate the onset and progression of CVD through the NLRP3 inflammasome pathway [165]. NLRP3, a key component of the innate immune system, is capable of sensing a variety of cellular stress and metabolic disorder signals [166]. The activation process of NLRP3 requires two steps, namely priming and activation [166]. Priming mainly involves NF-κB or other signaling pathways downstream of the Toll-like receptor, with the signals elevating the protein expression levels of NLRP3 and pro-IL-1β, and promoting post-translational modifications in NLRP3 [167]. Subsequently, the NLRP3 inflammasome can be activated by stimuli such as extracellular ATP. Activated NLRP3 oligomerizes and recruits the adapter protein apoptosis associated speck-like protein containing a CARD (ASC) [168]. ASC possesses a caspase recruitment domain (CARD) that recruits pro-caspase-1 through CARD-CARD interactions, thereby promoting caspase dimerization and activation [169]. Active caspase-1 can drive inflammatory signaling by recognizing and cleaving pro-IL-1β and pro-IL-18. In addition, it cleaves the pore-forming protein GSDMD, which mediates the onset of cellular pyroptosis and promotes the maturation and release of the pro-inflammatory cytokines IL-1β and IL-18 [166, 170].

The NLRP3 inflammasome is regarded as a link between stress and depression [171]. A study found that mice subjected to four weeks of chronic unpredictable mild stress exhibited depressive-like behavior and significantly elevated serum corticosterone levels. The inflammasome inhibitor, VX-765, effectively ameliorated depressive symptoms in mice with chronic unpredictable mild stress [172]. Psychological stress triggers a “dual signaling” activation mechanism of the NLRP3 inflammasome. Chronic psychological stress induces excessive GC secretion through activation of the HPA axis, thereby generating priming signals. GC acts on the GC receptor, and activated GC receptor signaling in turn significantly enhances NF-κB expression and promotes its nuclear translocation [173]. Furthermore, psychological stress promotes reactive oxygen species and ATP generation, with ATP activating the purinergic P2X7 receptor (P2X7R), triggering K^+^ efflux, which in turn promotes activation of the NLRP3 inflammasome and initiates the inflammatory cascade [171]. IL-1β produced after NLRP3 activation can induce depressive behaviors by inhibiting hippocampal neurons, disrupting the function of the HPA axis [174], inducing neuroinflammation [175], and other mechanisms [171, 174]. In addition to being involved in the onset of neuroinflammation and mood disorders, IL-1β is also extensively involved in the inflammatory mechanisms of CVD, such as atherosclerosis, hypertension, and myocardial injury [176].

Cell type differences exist in the cardiovascular activation of the NLRP3 inflammasome, with activation stronger in cardiac fibroblasts, endothelial cells, and macrophages, and weaker in cardiomyocytes. In myocardial infarction, the NLRP3 inflammasome in fibroblasts and endothelial cells drives IL-1β release and cellular pyroptosis, whereas only pyroptosis is mediated in cardiomyocytes. IL-1β released from fibroblasts and endothelial cells further recruits inflammatory macrophages and promotes activation of their NLRP3 inflammasome [177]. In addition, IL-1β and IL-18 drive the transformation of fibroblasts to myofibroblasts expressing α-smooth muscle actin, thus leading to excessive collagen deposition and promoting cardiac fibrosis [178, 179]. Inflammatory factors released from the activation of the NLRP3 inflammasome recruit macrophages and T-cells, triggering cardiomyocyte pyroptosis and cardiac inflammation. This ultimately leads to fibrosis, pathological cardiac remodeling, or even heart failure [180].

**Figure 7:**
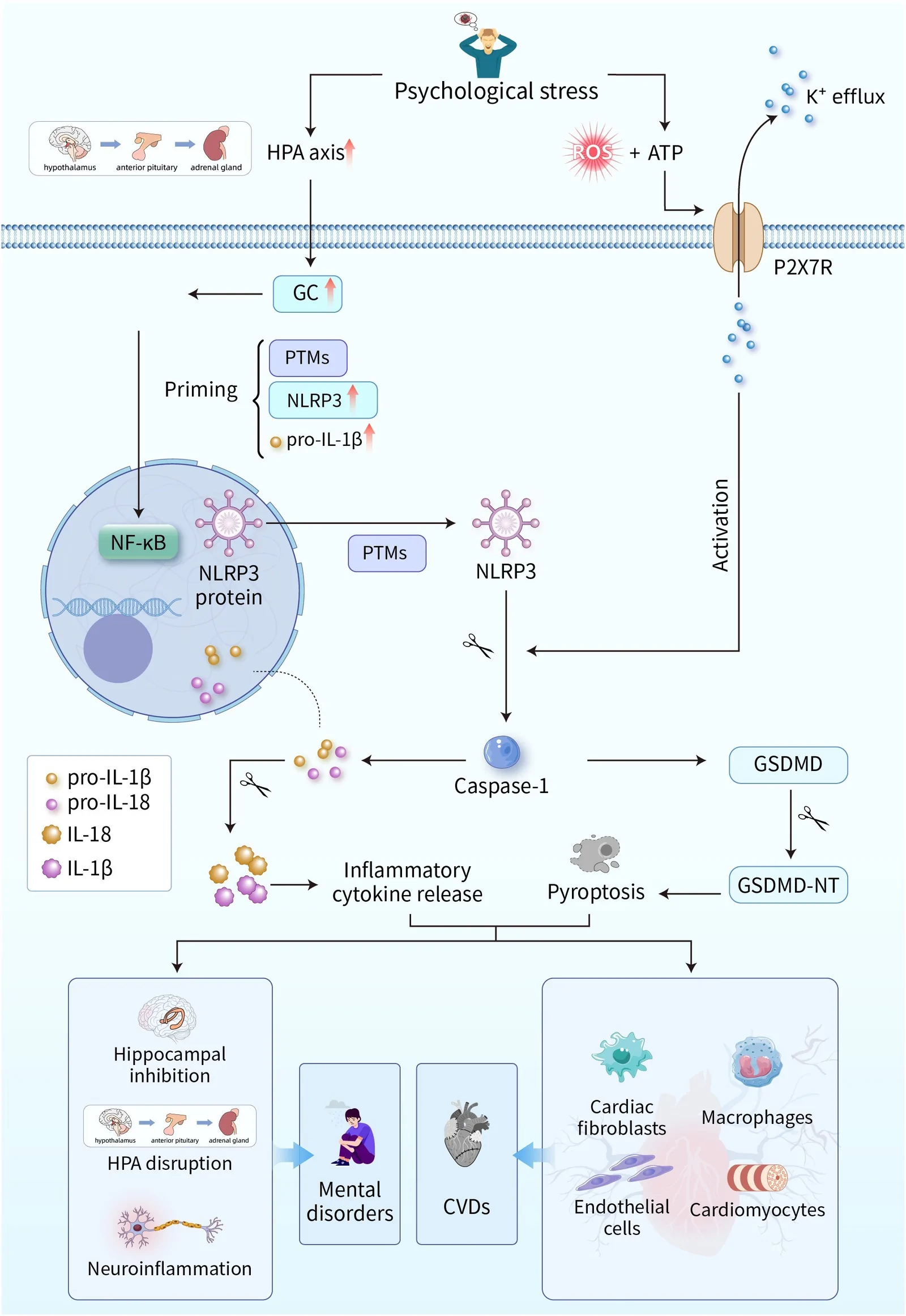
NLRP3 inflammasome pathway in PCIN and its dual effects on neuropsychiatric and cardiovascular systems. Psychological stress activates the HPA axis and induces excessive secretion of GC. This results in priming signals that activate NF-κB, upregulate the expression of NLRP3 proteins and pro-IL-1β, and promote the occurrence of PTMs in NLRP3. Psychological stress also induces ROS and ATP production, with ATP activating P2X7R and triggering K^+^ efflux, thereby promoting NLRP3 inflammasome activation. The activated NLRP3 recruits and activates caspase-1, which cleaves pro-IL-1β and pro-IL-18 to generate mature IL-1β and IL-18, and also cleaves GSDMD, leading to pyroptosis. This process can induce hippocampal inhibition, HPA axis disorders, and neuroinflammation, thus promoting the development of mental disorders; it can also act on cardiac fibroblasts, macrophages, endothelial cells, and cardiomyocytes, which promotes the onset and development of CVD. HPA: hypothalamic-pituitary-adrenal; GC: glucocorticoid; PTMs: post-translational modifications; NLRP3: NOD-like receptor protein 3; NF-κB: nuclear factor kappa B; ROS: reactive oxygen species; ATP: adenosine triphosphate; P2X7R: purinergic P2X7 receptor; IL-18: interleukin-18; IL-1β: interleukin-1β; GSDMD: gasdermin D; GSDMD-NT: Gasdermin D N-terminal; CVDs: cardiovascular diseases.

Chronic low-grade systemic inflammation, which is common in patients with mental disorders, is closely associated with NLRP3 activity, with the inflammatory environment aggravating pathological changes in the cardiovascular system and promoting the onset and development of CVD [181]. Recent evidence further supports NLRP3 as a potential shared inflammatory mechanism underlying the comorbidity of depression and CVD, although the available quantitative evidence for NLRP3 activation in depression is derived predominantly from animal studies [182, 183]. The bidirectional relationship between mental disorders and CVD may be manifested through NLRP3-mediated brain–heart pathways (Fig. 7). A study reported that cardiac pressure overload activates the central sympathetic nervous system through afferent neural signaling. This can prompt the release of ATP from sympathetic nerve endings, which binds to P2X7R on the surface of cardiac cells, triggering the assembly and activation of the NLRP3 inflammasome and the production of active IL-1β. The produced IL-1β ultimately induces cardiac inflammatory responses and cardiomyocyte hypertrophy, thereby triggering adaptive cardiac remodeling [178]. In the aforementioned study, subsequent NLRP3 activation, IL-1β production, and cardiac hypertrophy were significantly inhibited by the ablation of cardiac afferent nerves with capsaicin. The results exemplified the critical bidirectional regulatory relationship of brain–heart interactions in stress-induced cardiac remodeling. Notably, psychological stress may independently or synergistically trigger this inflammatory cascade response through activation of the sympathetic nervous system, which explains the association between psychological stress and cardiac disease at a mechanistic level [178]. In the heart–brain direction, reduced cerebral perfusion and intermittent hypoxia due to cardiac arrest and cardiac insufficiency create a microenvironment conducive to NLRP3 activation. This triggers the release of damage-associated molecular patterns (DAMPs) in the brain, which activate the NLRP3 inflammasome in microglial cells and consequently induce microglial cell pyroptosis and the release of IL-1β/IL-18. Ultimately, peripheral immune cells are recruited for infiltration, thus amplifying the neuroinflammatory cascade response and exacerbating brain damage. Such a mechanism may explain the high prevalence of anxiety and depression after cardiac events [184]. In view of the above mechanisms, the NLRP3 inflammasome has emerged as an important potential target for the treatment of psycho-CVD comorbidities [182]. Certain antidepressant drugs have been found to possess the ability to inhibit NLRP3-mediated inflammation [185, 186]. In addition, NLRP3 inhibitors such as MCC950, as well as monoclonal antibodies against IL-1β, have shown dual efficacy in alleviating both depressive-like behaviors and cardiovascular lesions in animal models [187–190].

## Potential intervention strategies based on the PCIN

**Figure 8:**
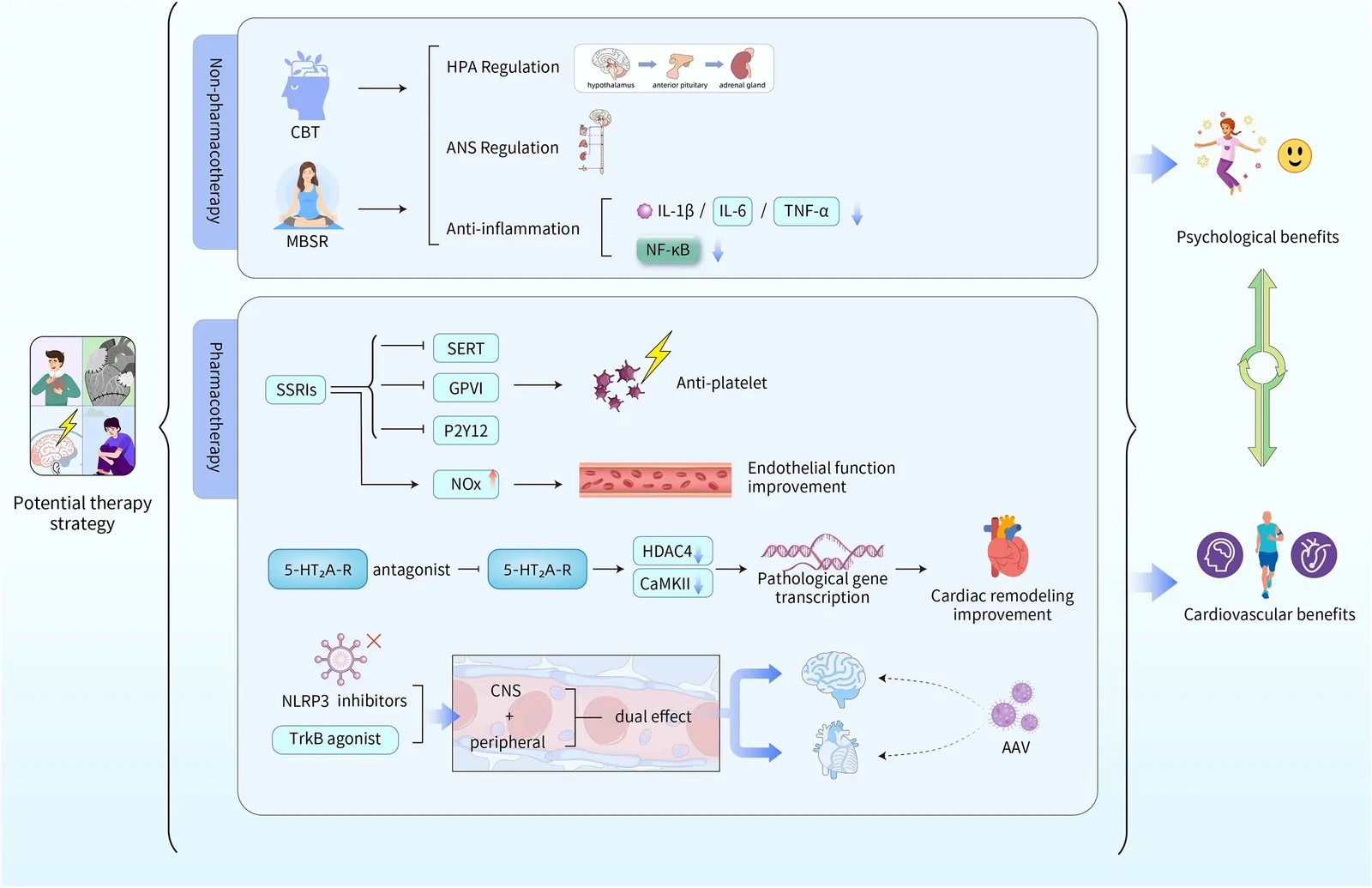
Potential therapeutic strategies targeting the PCIN and its molecular mechanisms. This figure demonstrates potential therapeutic strategies integrating heart–brain co-management concepts and their molecular mechanisms. Nonpharmacological treatments, including CBT and MBSR, produce both psychological and cardiovascular benefits by modulating the HPA axis, ANS, and anti-inflammatory effects. Pharmacological treatments include SSRIs, 5-HT_2_A-R antagonists, NLRP3 inhibitors, and TrkB agonists. SSRIs exert antiplatelet effects through inhibition of SERT, GPVI, and P2Y12 receptors, and improve endothelial function by increasing NOx. 5-HT_2_A-R antagonists block the downstream CaMKII/HDAC4 phosphorylation cascade, reduce pathological gene transcription, and improve cardiac remodeling. Ideal NLRP3 inhibitors and TrkB agonists should possess good blood-brain barrier permeability and exert dual neurocardiovascular protective effects on the CNS and peripheral tissues. AAV-mediated gene therapy has demonstrated good prospects for targeted therapies with greater precision. CBT: cognitive behavioral therapy; MBSR: mindfulness-based stress reduction; HPA: hypothalamic-pituitary-adrenal; ANS: autonomic nervous system; IL-1β: interleukin-1β; IL-6: interleukin-6; TNF-α: tumor necrosis factor-α; NF-κB: nuclear factor kappa B; SSRIs: selective serotonin reuptake inhibitors; SERT: serotonin transporter; GPVI: Glycoprotein VI; NOx: nitric oxide metabolite; 5-HT_2_A-R: 5-HT_2_A receptor; HDAC4: histone deacetylase 4; CaMKII: calcium/calmodulin-dependent protein kinase II; NLRP3: NOD-like receptor protein 3; TrkB: tropomyosin receptor kinase B; CNS: central nervous system; AAV: adeno-associated virus.

At present, the effects of mental health treatment on the prognosis of patients with CVD remain debated. Despite the high prevalence of anxiety and depression in the CVD population, previous meta-analyses have failed to confirm that psychotherapy improves primary clinical outcomes in patients with CVD [191]. However, psychological or pharmacological interventions significantly reduced rehospitalizations, emergency department visits, and all-cause mortality risk in patients with CVD and comorbid anxiety or depression, with combined interventions demonstrating superior clinical benefits [192]. Cardiac rehabilitation, an important component of comprehensive management strategies, has proven to be effective in improving the depressive symptoms of patients [16]. Based on the theoretical PCIN framework, future clinical interventions should aim to achieve a shift from the traditional organ-specific treatment model to a systemic health management model. By integrating the concepts of brain–heart co-management (Fig. 8), synergistic management of psychological and cardiovascular health can be realized to attain better clinical outcomes.

Nonpharmacological interventions, including cognitive behavioral therapy (CBT) and mindfulness-based stress reduction (MBSR), aid in improving symptoms of mental disorders, and positively affect cardiovascular health [193]. Successful CBT and MBSR treatment can reduce cortisol secretion in anxiety patients, contribute to normalization of abnormal HPA axis function [194, 195], and promote cortisol habituation of the HPA axis in response to repetitive stress. Such habituation is considered a marker of healthy HPA axis function and may reduce the risk of inflammation-related diseases such as CVD, but further studies are needed to validate the specific mechanisms [196]. MBSR intervention upregulates FK506-binding protein 5 methylation and reduces its expression [197], thus enhancing GC receptor sensitivity and improving the negative feedback regulation capacity of the HPA axis. Regarding ANS regulation, both CBT and MBSR can significantly improve HRV, enhance parasympathetic activity, and optimize the sympathetic-parasympathetic balance [198, 199], thereby contributing to psycho-cardiovascular regulation. In terms of immunomodulation, CBT and MBSR may influence pro-inflammatory signaling and cytokine levels [200], including IL-6, TNF-α, and IL-1β. In MBSR intervention studies conducted on elderly adults who experienced loneliness, no significant changes in inflammatory factors were observed, but MBSR was found to specifically downregulate NF-κB activity and its downstream pro-inflammatory gene expression [201]. Other behavioral interventions such as relaxation response training and cognitive-behavioral stress management demonstrated similar immunomodulatory effects [202, 203], suggesting that NF-κB pathway inhibition may be a common mechanism by which behavioral interventions exert anti-inflammatory effects.

SSRIs significantly reduce the risk of myocardial infarction and major adverse cardiovascular events in depressed patients with comorbid CVD. However, their influence on mortality remains to be elucidated [204]. SSRIs competitively inhibit platelet SERT, lowering platelet 5-HT levels and attenuating their aggregation response [205]. In addition to SERT-dependent pathways, certain SSRIs exert their antiplatelet effects by blocking the calcium and DAG guanine nucleotide exchange factor-1/Ras-proximate-1 (CalDAG-GEFI/Rap1) pathway, inhibiting the glycoprotein VI receptor [206], and downregulating P2Y12 receptor-mediated phosphorylation of Akt, glycogen synthase kinase-3β (GSK3β), p38 MAPK, and spleen tyrosine kinase (Syk) [207]. SSRIs also improve vascular endothelial function, with studies showing that paroxetine increased plasma nitric oxide metabolite levels and normalized reduced plasma nitric oxide metabolite levels in depressed patients [208]. However, the clinical benefits of SSRIs may be partially offset by their adverse metabolic effects, with paroxetine and sertraline use being associated with higher LDL-cholesterol levels [209]. Notably, heterogeneity exists in the cardiovascular effects of different SSRIs: certain drugs provide protective effects, whereas others may increase the risk of adverse events, and may promote the atherosclerotic process through pro-inflammatory mechanisms. Such variability depends on the specific type of drug, state of underlying disease, and individual genetic factors [210]. In light of these limitations, selective 5-HT_2_A-R antagonists have demonstrated therapeutic potential. For instance, M100907 has therapeutic potential in insomnia and depression, and can also be used to inhibit pathological hypertrophic gene transcription and improve cardiac remodeling [163]. Future studies are required to further elucidate the physiological functions of the 5-HT signaling system and 5-HT_2_A-R in the CNS and peripheral organs, and explore the mechanisms of their roles in inflammatory responses, endocrine regulation, and cardiovascular remodeling processes.

The NLRP3 inflammasome plays a key role in the stress–inflammation pathway linking mental disorders and cardiovascular injury, and its selective inhibitors have emerged as highly promising therapeutic targets [165]. Ideal NLRP3 inhibitors should possess good blood-brain barrier permeability to achieve dual regulation of central inflammation and peripheral cardiovascular inflammation. Although conventional inhibitors such as MCC950 exhibit cardioprotective effects, they possess limited CNS permeability [188]. Novel NLRP3 inhibitors such as NT-0796 and NT-0249 have shown good CNS permeability. In animal models, they have inhibited CNS inflammation while also improving systemic and cardiovascular inflammatory markers associated with obesity and metabolic disorders [211, 212]. However, no NLRP3 inhibitor has yet been approved for clinical application. Future studies should focus on the optimization of CNS permeability, safety assessment, and observation of long-term efficacy of such drugs, as well as the validation of their therapeutic potential in a variety of inflammation-related diseases.

The dual neuro-cardiovascular protective effects of the BDNF/TrkB signaling axis make it an ideal therapeutic target. 7,8-dihydroxyflavone, a small-molecule TrkB agonist, is orally bioavailable and can cross the blood-brain barrier. 7,8-dihydroxyflavone exerts neurotrophic effects by activating TrkB receptors to promote neuronal survival and repair, and is able to overcome the limitations of low BDNF delivery efficiency and short half-life. Therefore, it is expected to be a promising drug candidate for various neuropsychiatric disorders [213]. 7,8-dihydroxyflavone also protects the heart from ischemic injury, inhibits cardiac fibrosis, and improves cardiac function through multiple mechanisms, including regulation of the Akt, signal transducer and activator of transcription 3 (STAT3), AMP-activated protein kinase (AMPK), and ERK signaling pathways, inhibition of optic atrophy 1 (OPA1) proteolytic cleavage, and modulation of the Bmal1/Akt pathway [214–216].

Gene therapy has been widely used in animal models of CVDs and mental disorders [217, 218]. Among the existing viral vectors, adeno-associated viruses (AAVs) have thus emerged as ideal vectors due to their ability for sustained expression and tissue specificity [219]. AAV-mediated BDNF gene therapy has shown promising results in mental disorders. Fusing BDNF with the cell-penetrating peptides hemagglutinin-2 and transactivator of transcription, followed by delivery via intranasal administration using an AAV vector, significantly alleviated depressive-like behaviors in mice within 10 days [220]. In the cardiovascular field, AAV9 and AAV6 are the main vectors utilized because of their cardiotropic properties, and targeting can be further enhanced by combining with myocardial-specific promoters (e.g. cTnT and α-MHC) [221]. Although AAV-mediated gene therapy has made substantial progress in disease models such as heart failure and infarction, relevant studies on BDNF remain limited. Technical challenges related to neutralizing antibodies, optimization of delivery methods, and large-scale production will need to be addressed to advance the clinical translation of AAV-mediated gene therapy [219].

In summary, the current integrated brain–heart axis-informed intervention strategies mainly operate on two levels: (1) nonpharmacological interventions achieve dual protection by remodeling neuroplasticity and optimizing the neuroendocrine-immune network; (2) pharmacological and molecular-targeted therapies achieve precise interventions by modulating key signaling molecules (Fig. 8). However, existing management of psycho-cardiovascular risk is still mainly confined to the passive response mode of “disease-treatment”. Based on the theoretical PCIN framework, future therapeutic strategies should be shifted from the traditional single signaling pathway intervention approach to systematic regulation of a multipathway network. The paradigm shift from “passive treatment” to “active health” should be promoted, and an integrated intervention system covering bio-psycho-social-environmental dimensions should be established. In clinical practice, the importance of psychological monitoring and intervention should be emphasized, and mental health assessment should be incorporated into the routine clinical management of patients with CVD, particularly those with comorbid mental disorders. Building on this integrated framework, psycho-cardiovascular risk may be better characterized by considering psychosocial phenotypes alongside molecular, genetic, and physiological features. Such multidimensional profiling may help identify biologically distinct patient subgroups, refine cardiovascular risk assessment, and guide the selection of targeted interventions.

## Conclusion

The close association between psychological and psychiatric factors and CVD has been widely recognized [2]. The proposed PCIN framework aims to integrate the multilevel regulatory mechanisms of psycho-cardiovascular interactions. The identification of key signaling pathways and their regulatory molecules offers a theoretical basis for translational research on mental disorder–CVD comorbidity and may provide candidate biomarkers and therapeutic targets.

However, current evidence remains limited. First, most mechanistic studies rely on animal models, and their clinical translational potential and relevance to patients with mental disorders need to be further verified. Second, psycho-cardiovascular regulation involves multiple signaling pathways, whereas existing studies have mostly focused on a single pathway, with insufficient understanding of the signaling network across central and peripheral systems. In addition, high-quality randomized controlled trials assessing the long-term clinical effects of psychological interventions and their underlying molecular mechanisms in patients with CVD and comorbid mental disorders remain insufficient. The roles of individual genetic background and epigenetic modifications in psychological factor-mediated cardiovascular effects require further exploration.

Looking ahead, high-throughput screening and multiomics approaches are expected to enhance the understanding of the PCIN and identify molecular markers and therapeutic targets related to psycho-cardiovascular interactions. PCIN-related targeted therapeutic strategies also need to be evaluated across small- and large-animal models before clinical translation for mental disorder–CVD comorbidity. Novel therapeutic delivery systems, such as AAV-mediated gene therapy and exosome delivery platforms, together with combined central–peripheral intervention strategies, may provide opportunities for brain–heart axis-informed intervention. At the preventive level, molecular markers should be further explored to support dynamic assessment of psycho-cardiovascular status and individualized intervention.

The paradigm of psycho-cardiovascular risk prevention and intervention is shifting from the traditional organ-centered model to the psychological-physiological integration model. The incorporation of mental health assessments and psychological interventions into routine CVD management should be considered, particularly for patients with mental disorders or high psychological distress. Further analyses of the molecular mechanisms of the brain–heart axis, identification of biomarkers and therapeutic targets, and exploration of innovative intervention strategies are expected to promote synergistic improvement in cardiovascular and mental health. Ultimately, the goal is to construct a brain–heart axis-informed framework for psycho-cardiovascular prevention and intervention, with precision medicine serving as an important tool.
